# Supplementary material for: Extensive 5′-surveillance guards against non-canonical NAD-caps of nuclear mRNAs in yeast
Source: Nat Commun. 2020 Nov 2;11:5508. doi: 10.1038/s41467-020-19326-3 (PMC7606564; doi:10.1038/s41467-020-19326-3)
Supplement: Supplementary file 1 — Supplementary Information [file 41467_2020_19326_MOESM1_ESM.pdf]

Supplementary Information

Extensive 5'-surveillance guards against non-canonical NAD-caps of nuclear mRNAs in yeast

Zhang et al.

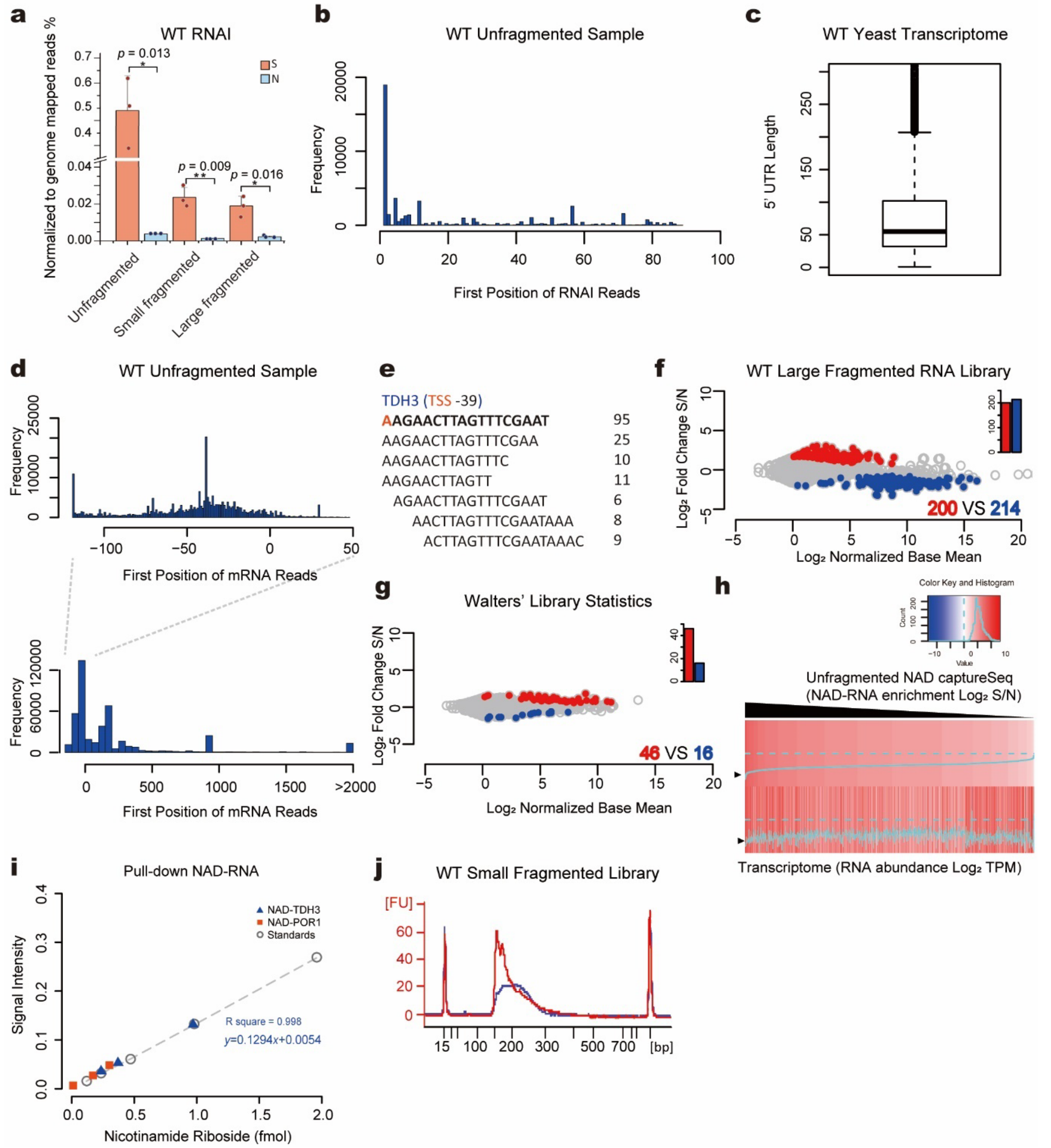

Supplementary Fig. 1 | NAD captureSeq library reference features and further analysis of the WT strain.

a, Method validation: Enrichment of spike-in NAD-RNAI (a regulatory RNA from *E. coli*) reads in WT NAD captureSeq. The height of the orange bars indicates the percentage of NAD-RNAI reads among total genome-mapped reads in the sample group (S, ADPRC fully-treated), and blue bar height indicates the same in the negative control group (N, minus ADPRC). Error bars represent the mean + standard deviation (sd), n=3. p values are denoted by asterisks: (*) p <0.05; (**) p <0.01; (Student's t test, one sided). This analysis revealed efficient enrichment of the synthetic pure NAD-RNA in all samples. Particularly high enrichment was observed in the unfragmented libraries.

b, Positions of the first nucleotide of spike-in RNAI-mapped reads in the WT sample, confirming the reliable identification of NAD-RNA 5'-ends from the NAD captureSeq data.

c, Distribution of 5' UTR lengths in *S. cerevisiae* according to published data [1]. The boxplot shows from bottom to top "minimum" (Q1-1.5 interquartile range (IQR, 25% to 75%)), first quartile (Q1, 25%), median (solid line, 50%), third quartile (Q3,75%), "maximum" (Q3+1.5IQR), and outliers (black dots). The sample size (n) is 4419.

d, Positions of the first nucleotide of genome-mapped reads in the WT unfragmented NAD captureSeq sample group. The 5' UTR region (-120 to +50, relative to the translation start site (TLS) as '0') is zoomed in in the upper panel, confirming the 5' UTR length distribution expected from the literature data visualized in Supplementary Fig. 1c.

e, Alignment of small RNAs (12-17 nt) with homology to TDH3 RNA observed in the WT unfragmented NAD captureSeq library. The red 'A' is the +1 nucleotide of the assumed transcription start site. The number on the right side of the sequence indicates the number of reads.

f, Enriched NAD-RNAs in the large fragmented NAD captureSeq libraries of the WT strain. All statistics parameters as in Fig. 1a. Performed in biological replicates, n=3.

g, Enriched NAD-RNA from Walters' published WT BY4742 yeast library [2]. All statistics parameters are as in Fig.1a.

h, Heatmap correlation between NAD-RNA enrichment (NAD captureSeq) and transcript abundance (transcriptome sequencing). Continuous line represents actual expression levels, while the dashed line serves as reference for comparison.

i, LC/MS standard curve for the quantification of pulled-down NAD-RNAs. The grey dashed line represents titrated nicotinamide riboside (NR, grey empty circles) as standard curve. Blue triangles denote the measured NR intensities for pulled-down NAD-TDH3 RNA, while red cubes represent NAD-POR1 RNA NR intensities. Performed in biological replicates, n=3.

j, Bioanalyzer electrophoresis of NADcapture Seq cDNA amplicon. The red line represents the cDNA amplicon of the NAD captureSeq sample group (+ADPRC treatment), while the blue line represents the negative control group (-ADPRC treatment). The x axis represents the cDNA length in base pairs (bp), while the y axis denotes the signal intensity. Peaks at 15 and 1500 bp represent size standards added for Bioanalyzer electrophoresis.

Source data are provided as a Source Data file.

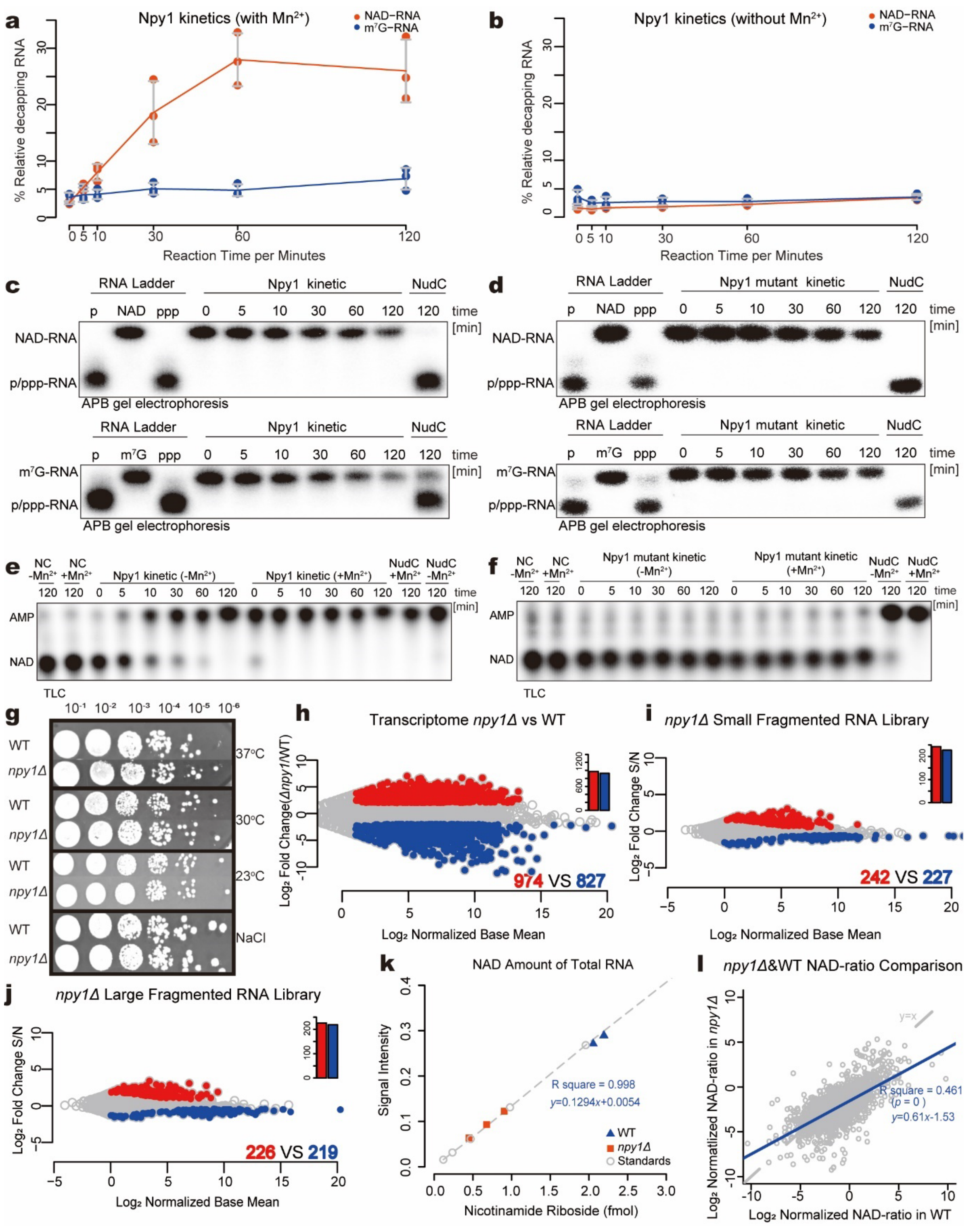

Supplementary Fig. 2 | Npy1 *in vitro* activity juxtaposed with WT and *npy1Δ* NAD captureSeq data sets.

a,b, Quantification curve of Npy1 kinetics with NAD- and m^{7}G-capped TDH3 RNA in the presence and absence of Mn^{2+} as shown in Fig. 3a and Supplementary Fig. 3c. Error bars represent mean ± sd. Three independent experiments were performed, n=3.

c, Npy1 WT kinetics of decapping NAD- and m^{7}G-capped RNA in the absence of Mn^{2+} *in vitro*. Otherwise, performed under identical conditions as in Fig. 3a.

d, Npy1 mutant (E276Q) kinetics in decapping NAD- and m^{7}G-capped RNA in the presence of Mn^{2+} *in vitro*.

e,f, Hydrolysis of NAD (yielding NMN and ATP), catalyzed by WT Npy1 and mutant (E276Q) Npy1 *in vitro*. ^{32}P-NAD was treated with the respective enzyme in the presence of 2 mM Mg^{2+} and 1 mM Mn^{2+} and reaction mixtures separated by thin layer chromatography (TLC, NH_4OAc/EtOH 4:6).

g, Growth phenotype comparison between WT and *npy1Δ* strain under different conditions. Cells were spotted in 10-fold serial dilutions starting from OD_{600} = 1. The cells were cultured in normal YPD medium at 30 °C, while the NaCl set was additionally supplemented with 0.5 M NaCl.

h, Analysis of expression level changes of transcripts upon removal of Npy1 by transcriptome sequencing. 7620 different transcripts were analyzed and are represented as dots. Red dots: up-regulated transcripts (fold change >1.414, normalized base mean >1, p <0.05 (DEseq2, negative binomial distribution)), blue dots: down-regulated transcripts (fold change <0.707, normalized base mean >1, p <0.05). Performed in biologically independent replicates, n=3.

i, Enriched NAD-RNAs in the small fragmented NAD captureSeq library of the *npy1Δ* strain. Other parameters as in Fig. 1a. Performed in biologically independent replicates, n=3.

j, Enriched NAD-RNAs in the large fragmented NAD captureSeq library of the *npy1Δ* strain. Other parameters as in Fig. 1a. Performed in biologically independent replicates, n=3.

k, LC/MS standard curve with total NAD-RNA. Same analysis as Supplementary Fig. 1i, but using total RNA for the measurement. Performed in biologically independent replicates, n=3.

l, Relative RNA NAD-modification ratio trend from integration of NADcaptureSeq, transcriptome, and LC-MS data. Grey dashed line equals y=x as reference, while the blue solid line represents the linear regression of the experimental data: $\textit{NAD-ratio}_{\Delta Npy1}=k^{*}\ \textit{NAD-ratio}_{WT}+c$ (k denotes the slope, while c corresponds to the intercept).

Source data are provided as a Source Data file.

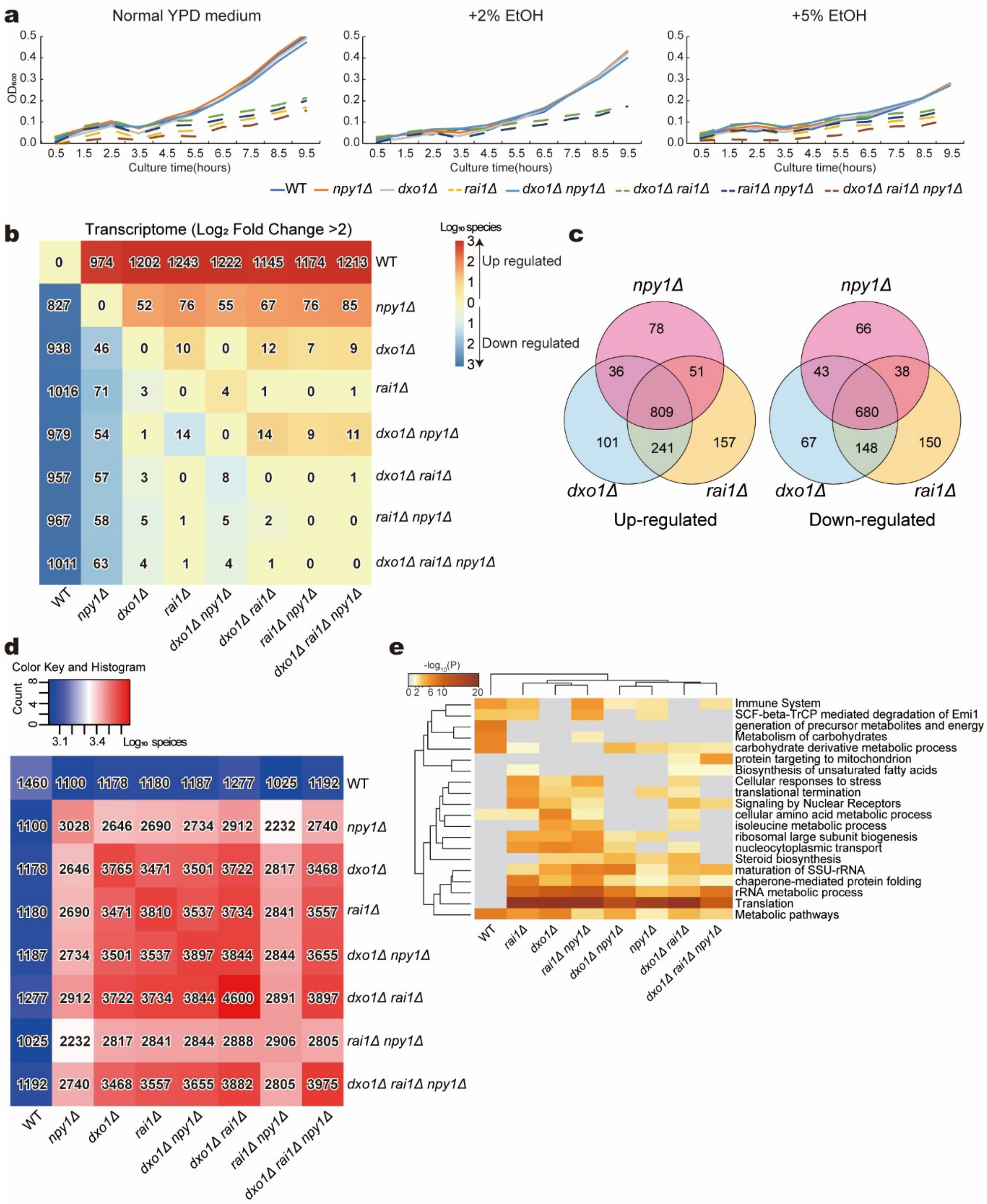

Supplementary Fig. 3 | Detailed comparative analysis of yeast deletion mutants.

a, Time course of the cell density (OD_{600}) for WT and all deletion mutants. The mutant strains harboring a *RAI1* gene deletion are indicated by dashed lines while all other strains are plotted as continuous lines. The cells were incubated in YPD medium on 96 well plates at 30 °C under agitation. The cell density was inferred based on the optical density at 600 nm at the identical time points.

b, Heatmap of the differential expression assessed by transcriptome sequencing, comparing the number of up-regulated (red, above the “0 0 0” diagonal) and down-regulated (blue, below the “0 0 0” diagonal) transcripts between two respective strains. The number of RNA species was log_{10}-transformed and resulting values color-

coded. Example: Comparison of *npy1*Δ and WT strain yields 974 species exhibiting up-regulation and 827 species exhibiting down-regulation.

c, Venn diagrams showing the intersection of up-/down- regulated RNA species between the three single-deletion strains by transcriptome sequencing. Biologically independent samples n=3.

d, Heatmap of the intersection of enriched NAD-RNAs assessed by NAD captureSeq. The number of RNA species that overlap between two strains was $\log_{10}$ transformed and scaled by color intensity. Each group comprises biologically independent replicates, n=3.

e, Heatmap of functional clustering of the top 250 enriched NAD-RNA species for WT and all 7 deletion strains by gene ontology (GO) terms. The color intensity represents the $\log_{10}$ transformation of the p value. Each group comprises biologically independent replicates, n=3.

Source data are provided as a Source Data file.

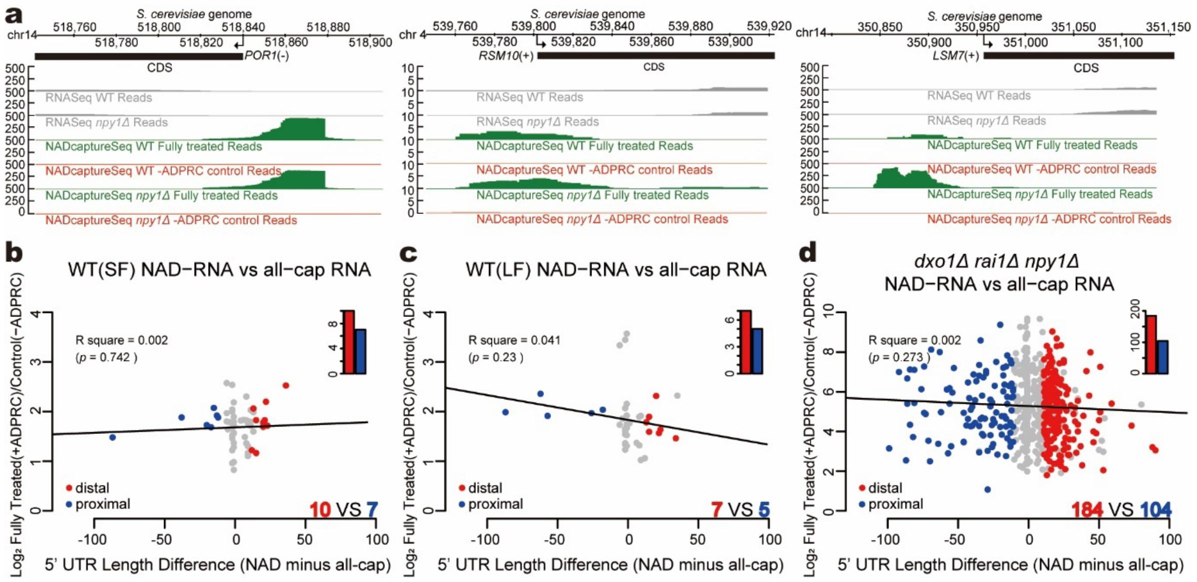

Supplementary Fig. 4 | Genome-wide 5'-transcript leader features of NAD-RNAs.

a, Comparison of the read profiles of WT and *npy1Δ* samples. Aligned reads of NAD captureSeq and transcriptome sequencing were normalized as RPM and visualized in the IGB. Green patterns represent accumulated reads in the fully treated sample group (+ADPRC), while the red traces were derived from the -ADPRC negative control in NAD captureSeq data sets (unfragmented libraries). The grey traces represent the read distribution of transcripts from transcriptome sequencing.

b, Identical experimental and analytical procedures as in Fig. 6c, but using the small fragmented WT NAD captureSeq libraries.

c, Identical experimental and analytical procedures as in Fig. 6c, but using the large fragmented WT NAD captureSeq libraries.

d, Identical experimental and analytical procedures as in Fig. 6c, but using the *dxo1Δ rai1Δ npy1Δ* triple knockout NAD captureSeq libraries.

Source data are provided as a Source Data file.

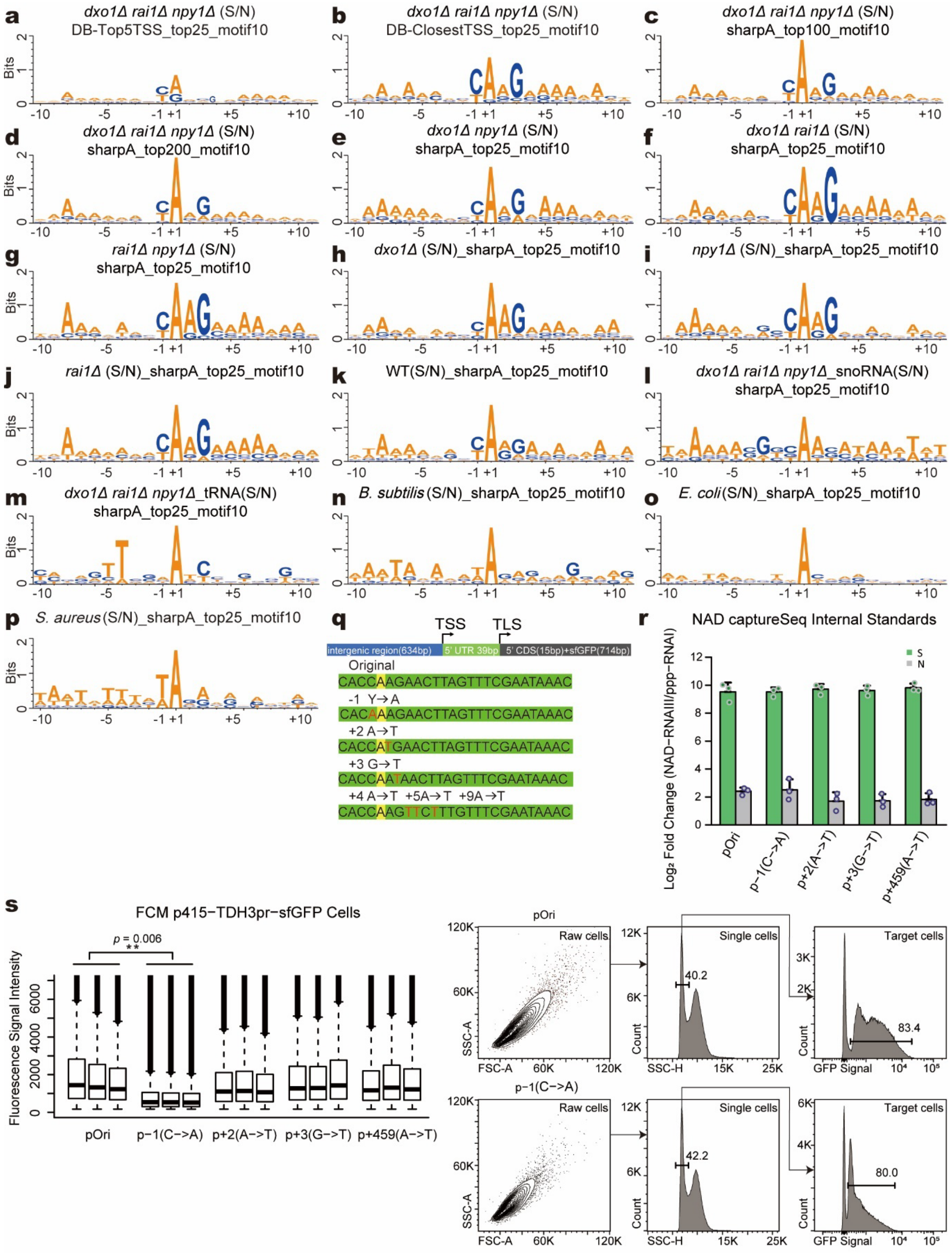

Supplementary Fig. 5 | Analysis and comparison of key promoter motifs in other mutants and organisms.

a-m, Motif analysis of the -10 to +10 region around the TSS based on NAD captureSeq data as in Fig. 7a for different selected populations and mutant strains. Top 100 and top 200 represent 100 and 200 most enriched NAD-mRNA species, respectively. Enriched NAD-capped snoRNA and tRNA are represent snoRNA(S/N) and tRNA(S/N), respectively.

n-p Motif analysis of the top 25 enriched NAD-mRNA species in *B. subtilis* [3], *E. coli* [4], and *S. aureus* [5], respectively. All parameters as in Fig. 7a.

q, Scheme illustrating the *TDH3* gene promoter and relevant mutations. The yellow ‘A’ is the TSS and referenced as +1. The red letters highlight mutations.

r, Spike-in NAD-RNAIII and ppp-RNAI served as the standards for TDH3 RNA NAD-ratio quantification. Statistics parameters as in Fig. 7c. Error bars represent the mean ± sd. Performed in biologically independent replicates, n=3.

s, Flow cytometry analysis and analysis of GFP expression levels of the yeast strains shown in Fig. 7d, using three independent clones. The solid lines in the boxplot represent the median fluorescence signal intensity for each group (>75000 events in pOri, p-1, p+2, p+3, and p+459). The median values of each group were compared, p value is denoted by asterisks: (**) p <0.01 (Student’s t test, one-sided). Outliers above 7000 were not shown and omitted. The layout of the boxplot is similar to Supplementary Fig. 1c. On the right panel, 30000 collected cells from the pOri and p-1 strain were first subdivided into single cell populations by a SSC-H gate. Subsequently, GFP signal intensity was measured after removal of background signal by GFP (488-E-A). The subgroup percentage of gated events is denoted beside the indicated range.

Source data are provided as a Source Data file.

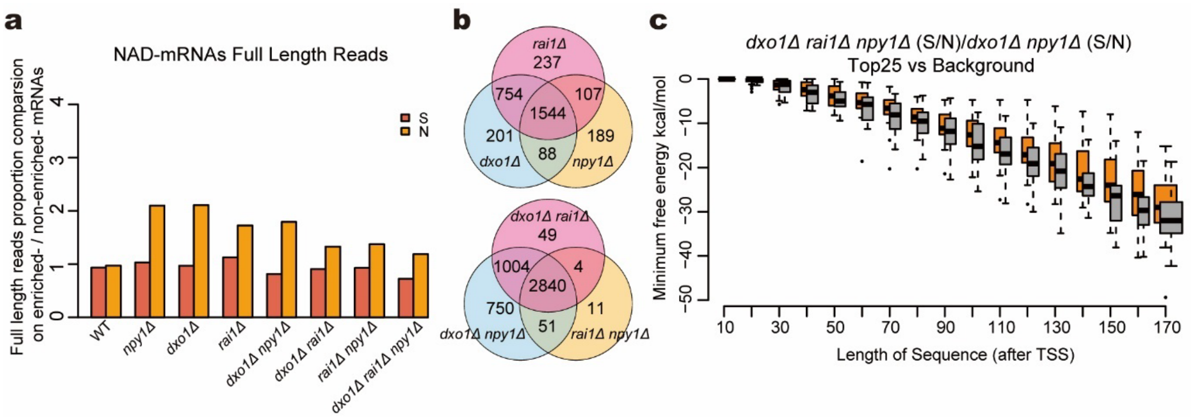

Supplementary Fig. 6 | Yeast decapping enzymes target different NAD-RNA species.

a, Ratio of full length reads of mRNAs in NAD captureSeq libraries. The bar heights indicate the proportion of full length reads, obtained from enriched NAD-RNA species divided by the proportion obtained from non-enriched RNA species. The red bars represent the percent ratios in NAD captureSeq sample group (+ADPRC, S), while the orange bars represent the percent ratios in the NAD captureSeq negative control group (-ADPRC, N).

b, Venn diagrams showing the intersection of enriched NAD-RNA species identified in the three single mutant (top) and double mutant strains (bottom, unfragmented libraries).

c, Prediction of TL folding energy using RNAfold [6] as a function of the assumed transcript length. The orange bars represent the predicted minimum free energy of the top 25 highest-enriched NAD-RNAs when comparing the *dxo1Δ rai1Δ npy1Δ* triple knockout (S/N) with the *dxo1Δ npy1Δ* double knockout (S/N), while the grey bars refer to background RNAs (no significant NAD enrichment change (0.707 < *dxo1Δ rai1Δ npy1Δ* (S/N)/ *dxo1Δ npy1* (S/N) < 1.414) between the two strains). The layout of the boxplot is identical to Supplementary Fig. 1c.

Source data are provided as a Source Data file.

Supplementary Table 1: Reagents and Resources

REAGENT or RESOURCE	SOURCE	IDENTIFIER
**Yeast BY4742 Strains**		s
WT / BY4742 (*MATα his3Δ1 leu2Δ0 lys2Δ0 ura3Δ0*)	Lab stock [7]	N/A
*npy1*Δ (BY4742 *npy1::kanMX4*)	Lab stock [8]	N/A
*dxo1*Δ (BY4742 *dxo1::natNT2*)	This study	N/A
*rai1*Δ (BY4742 *rai1::hphNT1*)	This study	N/A
*dxo1*Δ *npy1*Δ (BY4742 *dxo1::natNT2 npy1::kanMX4*)	This study	N/A
*rai1*Δ *npy1*Δ (BY4742 *rai1::hphNT1 npy1::kanMX4*)	This study	N/A
*dxo1*Δ *rai1*Δ (BY4742 *dxo1::natNT2 rai1::hphNT1*)	This study	N/A
*dxo1*Δ *rai1*Δ npy1Δ (BY4742 *dxo1::natNT2 rai1::hphNT1 npy1::kanMX4*)	This study	N/A
**Yeast ESM356-1 Strains**		
wt (*MATa ura3-52 leu2Δ1 his3Δ200 trp1Δ63*)	Lab stock [9]	
YDK581-1: tdh3prΔtdh3Δ (*wt tdh3prΔtdh3Δ::hphNT1*)	This study	N/A
pOri plasmid in YDK581-1	This study	N/A
p-1 plasmid in YDK581-1	This study	N/A
p+2 plasmid in YDK581-1	This study	N/A
p+3 plasmid in YDK581-1	This study	N/A
p+459 plasmid in YDK581-1	This study	N/A
p415GalL plasmid in YDK581-1	This study	N/A
**Yeast C-SWAT mNeonGreen (mNG-I) Strains**		
Dxo1-mNeonGreen *(MatA YDR370C::mNeonGreen leu2Δ0::GAL1pr-NLS-SceI-natNT2 can1Δ::STE2pr-SpHIS5 lyp1Δ::STE3pr-LEU2 his3Δ1 ura3Δ0* ***met15Δ0 or MET15****)*	Lab stock [10]	N/A
Npy1-mNeonGreen*(MatA YGL067W::mNeonGreen leu2Δ0::GAL1pr-NLS-SceI-natNT2 can1Δ::STE2pr-SpHIS5 lyp1Δ::STE3pr-LEU2 his3Δ1 ura3Δ0* ***met15Δ0 or MET15****)*	Lab stock [10]	N/A
Rai1-mNeonGreen*(MatA YGL246C::mNeonGreen leu2Δ0::GAL1pr-NLS-SceI-natNT2 can1Δ::STE2pr-SpHIS5 lyp1Δ::STE3pr-LEU2 his3Δ1 ura3Δ0* ***met15Δ0 or MET15****)*	Lab stock [10]	N/A
**Yeast BY4741 Strains**		
wt (*MATa his3Δ1 leu2Δ0 met15Δ0 ura3Δ0*)	Lab stock [7]	N/A
YDK53-7: Membrane TagBFP (*wt his3Δ::GPDpr-TagBFP-PLCdelta-PH2-GPDterm*)	unpublished (D. Kirrmaier)	N/A
**Yeast ADPRC Strain**		
Pichia pastoris GS115 (pPICZαA/CYCLASE-2)	H.C. Lee Lab, Peking University [11]	N/A
**Chemicals, Peptides, and Recombinant Proteins**		
Aqua Phenol	Carl Roth	A980.3
Aqua-Phenol/ Chloroform/ Isoamyl alcohol	Carl Roth	X985.2
Acrylamide/Bis-solution 40 % (19:1)	Carl Roth	A516.1
Ammonium persulfate	Sigma-Aldrich	A3678
N,N,N’,N’-Tetramethyl ethylenediamine	Sigma-Aldrich	1.10732

(TEMED)		
Formic acid UPLC-MS optigrade®	Promochem	SO-9679-B001
Acetonitrile (UPLC/MS grade)	Fluka	65709
Bacto™ peptone	Becton Dickinson	211820
Bacto™ yeast extract	Becton Dickinson	212720
D(+)-glucose monohydrate	Carl Roth	6887
Difco™ agar	Becton Dickinson	214530
Geneticin (G418) sulfate	Santa Cruz Biotechnology	sc-29065B
Hygromycin B, 50 mg/mL	Thermo Scientific	10687010
Nourseothricin, clonNAT	Werner BioAgents	5.0000
Lithium acetate dihydrate	Sigma-Aldrich	L4158
Phenol:Chloroform:Isoamylalcohol (25:24:1)	Carl Roth	A156
Poly(ethylene glycol), 3350	Sigma-Aldrich	88276
Salmon sperm	Invitrogen	AM9680
Trizma® base	Sigma-Aldrich	T1503
Ethylenediaminetetraacetic acid (EDTA)	Gerbu	1034
dNTP sets, 100 mM each	Genaxxon	M3015
Taq DNA polymerase, 5 U/µL	New England Biolabs	M0273
GeneRuler 1Kb, ready-to-use	Thermo Scientific	SM0313
AccuPrime™ Pfx DNA polymerase, 2.5 U/µL	Invitrogen	12344
Zymolase 100T, 10 mg/mL	US biological	Z1005
Potassium acetate	Sigma-Aldrich	P1190
Adenine	Sigma-Aldrich	A8626
Arginine	Sigma-Aldrich	A5006
Aspartic acid	Sigma-Aldrich	A9256
Histidine	Sigma-Aldrich	H8000
Isoleucine	Sigma-Aldrich	I2752
Leucine	Sigma-Aldrich	L8000
Lysine	Sigma-Aldrich	L5501
Methionine	Sigma-Aldrich	M9625
Phenylalanine	Sigma-Aldrich	P2126
Threonine	Sigma-Aldrich	T8625
Tryptophan	Sigma-Aldrich	T8941
Tyrosine	Sigma-Aldrich	T3754
Uracil	Sigma-Aldrich	U0750
Valine	Sigma-Aldrich	V0500
DNase I recombinant, RNase-free	Sigma-Aldrich	4716728001
T4 Polynucleotide Kinase(T4 PNK)	Thermo Scientific	EK0031
ADP-Ribosylcyclase (ADPRC)	This study	N/A
4-pentyn-1-ol	Sigma-Aldrich	302481-5G
THPTA	Lab stock [12]	N/A
Azide-PEG3-biotin conjugate	Sigma-Aldrich	762024
Mobicol Classic columns	MoBiTec	M1003
Filter (small) 10 µm pore size	MoBiTec	M2110
Streptavidin Sepharose High Performance	GE Healthcare	GE17511301
Hydrophilic Streptavidin Magnetic Beads	New England Biolabs	S1421S

Albumin, Acetylated from bovine serum	Sigma-Aldrich	B8894
Gibco™ phosphate buffered saline (PBS)	Thermo Scientific	10010023
T4 RNA Ligase	Thermo Scientific	EL0021
T4 RNA Ligase 2, truncated K227Q	New England Biolabs	M0351L
SuperScript III reverse transcriptase	Thermo Scientific	18080085
SuperScript IV reverse transcriptase	Thermo Scientific	18090010
Exonuclease I (E. coli)	New England Biolabs	M0293L
Terminal Deoxynucleotidyl Transferase	Thermo Scientific	EP0162
T4 DNA Ligase	Thermo Scientific	EL0013
Q5 Hot Start High-Fidelity DNA Polymerase	New England Biolabs	M0493S
NEBNext Ultra II Q5 Master Mix	New England Biolabs	M0544S
NEBNext Multiplex Oligos for Illumina (Index Primers Set 1)	New England Biolabs	E7335L
NEBNext Multiplex Oligos for Illumina (96 Index Primers)	New England Biolabs	E6609S
Agencourt RNAClean XP	Beckman Coulter	A63987
E. coli NudC	Lab stock [4]	N/A
Alkaline Phosphatase	Sigma-Aldrich	P5931
d4-riboside nicotinamide	Toronto Research Chemicals	N407772
Zeocin Selection Reagent	Thermo Scientific	R25001
IPTG	Sigma-Aldrich	I5502
Zellu Trans 5.0V MWCO 5000	Carl Roth	E894.1
HiTrap SP HP cation exchange chromatography column	GE Healthcare	17115101
HisTrap HP histidine-tagged protein purification columns	GE Healthcare	17524701
HiTrap Heparin HP affinity columns	GE Healthcare	17040601
Sephacryl 16/60 S-200 High Resolution column	GE Healthcare	17511500
Amicon centrifugal filter 0.5mL 10 kDa MWCO	Merck	Z740202
Amicon centrifugal filter 15 mL 10 kDa MWCO	Merck	UFC901024
0.45 µm filter	Millipore	HAWP04700
Pierce BCA protein assay	Life Technologies	23252
Npy1	This study	N/A
Npy1 (E276Q)	This study	N/A
Dcp2	This study	N/A
$MgCl_2$	Sigma-Aldrich	M2670
$MnCl_2$	Sigma-Aldrich	M1787
T7 RNA polymerase	Lab stock	N/A
RiboLock RNase Inhibitor	Thermo Scientific	EO0381
RNA Century Marker Templates	Thermo Scientific	AM7780
Decade Markers System	Thermo Scientific	AM7778
ScriptCap m7G Capping System	CellScript	C-SCCE0625
Vaccinia Capping System	New England Biolabs	M2080S
RNA 5’ end polyphosphatase	Epicentre	RP8092H
Xrn-1	New England Biolabs	M0338L
Acryloylaminophenyl boronic acid	Lab stock [13]	N/A
DC-Fertigfolie ALUGRAM Xtra SIL G / UV254	Carl Roth	3879.1
SsoAdvanced Universal SYBR Green Supermix	Bio-Rad	1725272
Phenylmethylsulfonyl fluoride	Sigma-Aldrich	10837091001

Glass beads, ~0.5 mm	Sigma-Aldrich	Z250465
Medical Millex-HP Syringe Filter Unit, 0.45 µm	Millipore	SLHPM33RS
HiPrep 26/10 Desalting Columns (Sephadex G-25 Fine resin)	GE Healthcare	17508701
Micrococcal Nuclease	New England Biolabs	M0247S
Ethylene glycol-bis(2-aminoethylether)-N,N,N′,N′-tetraacetic acid (EGTA)	Sigma-Aldrich	E3889
Creatine phosphate	Sigma-Aldrich	10621714001
Creatine Phosphokinase from rabbit muscle	Sigma-Aldrich	C3755
Amino Acid Mixtures	Promega	L4461
RNasin Ribonuclease Inhibitors	Promega	N2611
Renilla-Glo Luciferase Assay System	Promega	E2710
Bright-Glo Luciferase Assay System	Promega	E2610
ULTRAhyb Ultrasensitive Hybridization Buffer	Thermo Scientific	AM8670
Whatman Nytran SuPerCharge nylon blotting membrane	Sigma-Aldrich	WHA10416294
TMT10plex Isobaric Label Reagent Set, 1 x 0.8 mg	Thermo Scientific	90110
Trypsin (sequencing grade)	Promega	V5111
BamHI-HF	New England Biolabs	R3136S
NdeI	New England Biolabs	R0111S
EcoRV-HF	New England Biolabs	R3195S
HindIII-HF	New England Biolabs	R3104S
SacI-HF	New England Biolabs	R3156S
XbaI	New England Biolabs	R0145S
α-^{32}P-ATP	Hartmann Analytic	FP-207
α-^{32}P-CTP	Hartmann Analytic	FP-809
γ-^{32}P-ATP	Hartmann Analytic	FP-301
^{32}P-NAD	Perkin Elmer	NEG023X
**Critical Commercial Assays**		
Ribo-Zero Gold rRNA Removal Kit (Yeast)	Illumina	MRZY1324
NEBNext Ultra II Directional RNA Library Prep Kit for Illumina	New England Biolabs	E7760S
NAD/NADH Quantitation Kit	Sigma-Aldrich	MAK037
**Oligonucleotides**		
Random hexamers	Jena Bioscience	PM-301
DNA Oligos listed in Supplementary Table 2	Integrated DNA Technologies and Sigma-Aldrich	https://www.idtdna.com www.sigmaaldrich.com
Pre-adenylated 3' adapter	Lab stock [12]	N/A
**Recombinant DNA**		
pDisplay-AP-CFP-TM	Addgene	20861
pET-28c-NudC	Lab stock [4]	N/A
pET-28c-NudC-V157A-E174A-E177A-E178A	Lab stock [4]	N/A
pET-28a-NPY1	This study	N/A
pET-28a-NPY1 mutant	This study	N/A
pET-28a-hDcp2	Addgene	72214
pBK77N_hybSV40_Luc2_empty_BGH_*Renilla*_CMV	D. Grimm Lab, Heidelberg University	N/A
pFK-DVs-R2A	[14]	N/A
pMaM5 (p415GPD-sfGFP)	Lab stock, not published	

pDisplay-AP-CFP-TM-5UTR-Luciferase	This study	N/A
pFA6a-hphNT1	Lab stock [15]	N/A
pFA6a-natNT2	Lab stock [15]	N/A
pOri (modified p415GPD, TDH3 promoter, fused with sfGFP)	This study	N/A
p-1 (mutated pOri TSS -1 C to A)	This study	N/A
p+2 (mutated pOri TSS +2 A to T)	This study	N/A
p+3 (mutated pOri TSS +3 G to T)	This study	N/A
p+459 (mutated pOri TSS +4&+5&+9 A to T)	This study	N/A
p415GalL	[16]	N/A
**Software and Algorithms**		
ImageQuant-8.1	GE Healthcare	29-0006-05
Bowtie-1.1.2	[17]	https://sourceforge.net/projects/bowtie-bio/files/bowtie/1.1.2/
Bwa-0.7.13	[18]	https://sourceforge.net/projects/bio-bwa/files/bwa-0.7.10.tar.bz2
samtools-0.1.13	[19]	https://sourceforge.net/projects/samtools/files/samtools/0.1.13/
Integrated Genome Browser	[20]	https://bioviz.org/download.html
HTSeq-0.6.0	[21]	https://pypi.python.org/pypi/HTSeq/0.6.0
DESeq2-1.4.5	[22]	https://bioconductor.org/packages/2.14/bioc/src/contrib/DESeq2_1.4.5.tar.gz
Bioconductor-2.14	Bioconductor	https://bioconductor.org/packages/2.14
David-6.7	[23]	https://david-d.ncifcrf.gov/
FGNet-3.16.0	[24]	https://bioconductor.org/packages/release/bioc/src/contrib/FGNet_3.16.0.tar.gz
Mascot-2.2.07	[25]	https://omictools.com/mascot-server-tool
MEME-5.0.3	[26]	http://meme-suite.org/meme-software/5.0.3/meme-5.0.3.tar.gz
WegLogo-3	[27]	http://weblogo.threeplusone.com/create.cgi
Metascape	[28]	http://metascape.org
Cytoscape-3.7.0	[29]	https://cytoscape.org/download.html
limma- 3.38.3	[30]	https://bioconductor.org/packages/release/bioc/html/limma.html
vsn-3.50.0	[31]	https://bioconductor.org/packages/release/bioc/html/vsn.html
Flowing Software-2.5.1	http://flowingsoftware.btk.fi/	http://flowingsoftware.btk.fi/download.php
Python-2.7.12	http://www.python.org	https://www.python.org/ftp/python/2.7.12/Python-2.7.12.tgz
Python-3.6.0	http://www.python.org	https://www.python.org/ftp/python/3.6.0/Python-3.6.0.tgz
perl-5.22.1	http://www.perl.org	https://www.cpan.org/src/5.0/perl-5.22.1.tar.gz
R-3.1.0	http://www.R-project.org	https://cran.r-project.org/bin/windows/base/old/3.1.0/
R-3.5.1	http://www.R-project.org	https://cran.r-project.org/bin/windows/base/old/3.5.1/
**Other**		
Illumina NextSeq 500	EMBL	https://www.embl.de

Proteomics UPLC-MS	EMBL	https://www.embl.de
LightCycler 480 Instrument II	Roche	5015278001
FP-6500/6600 Spectrofluorometer	Jasco	B016860822
Thermocycler C1000 Touch	Bio-Rad	1851148
Typhoon FLA 9500	GE Healthcare	4722596B
Waters Xevo TQ-S (UPLC/MS/MS)	Waters	N/A

Supplementary table 2: DNA oligonucleotides used in this study.

Name	Sequence (5'-to-3' direction)	Used for
hph_JE345fw	GGCTGTGTAGAAGTACTCGCCG	KO confirmation
Kan_K2	GTCAAGACTGTCAAGGAGGG	KO confirmation
Kan_K3	CGCCTCGACATCATCTGCCC	KO confirmation
Nat_fw1	CTAATCTCGAGGCGAATTTC	KO confirmation
DXO1_S1	GATGATTTTATGTCAAAGTTTTCTTTCTAGCCAAAC TCAGTTATGCGTACGCTGCAGGTCGAC	DXO1 KO generation
DXO1_S2	ATGAAATATTTCTCGGAAAAGTATTATATATTATAC ACGTCTCTAATCGATGAATTCGAGCTCG	DXO1 KO generation
DXO1_5v	GTTGTCTCTAATATCATCCG	KO confirmation
DXO1_3v	CCGAACTATTATTGATTTCCC	KO confirmation
NPY1_5v	TTGCCACATCGCTTCTTG	KO confirmation
NPY1_3v	CTCTGCATCGCCTGACCT	KO confirmation
RAI1_S1	GTGAAAGAATAGCGAAATATTAGACCAACATAGTG TATCCCAATGCGTACGCTGCAGGTCGAC	RAI1 KO generation
RAI1_S2	CGTCGATGAGGATATGCGCAGGAAAGACATAAAG GAATATTGTTAATCGATGAATTCGAGCTCG	RAI1 KO generation
RAI1_5v	GGCCAGATTAACCCATTTAG	KO confirmation
RAI1_3v	GACAAAGCATTTGGTTGAGC	KO confirmation
F_T7_NAD-RNAI	AATAATACGACTCACTATTACAGTATTTGGTATCTG CGCTCT	NGS spike-in control
R_NAD-RNAI	ACAAAAAAACCACCGCTACCAGCGGTGGTTTGTTT GCCG	NGS spike-in control
biotin_POR1_probe	TGCTTTGGGTACGCTTGGCTGTTTCGTGTT-TEG-biotin	RNA pull-down
biotin_TDH3_probe1	TTATGTGTGTTTATTCGAAACTAAGTTCTT-TEG-biotin	RNA pull-down
biotin_TDH3_probe2	CTACCGAAACCGTTAATAGCAACTCTAACC-TEG-biotin	RNA pull-down
biotin_RDN5-1_probe	CGGGAAACGGTGCTTTCTGGTAGATATGGCCGCA ACC	RNA pull-down
F_TDH3_5UTR-qE	GAACTTAGTTTCGAATAAACACACA	qPCR for transcripts & ratio
R_TDH3_5UTR-qE	TCTCATGACCAATCTACCG	qPCR for transcripts & ratio
F_TDH3_TLS-qE	TTGGTCATGAGAATTGCTTTGT	qPCR for transcripts
R_TDH3_TLS-qE	GCAGCGTAGTCGTTGGTGAT	qPCR for transcripts
F_TDH3_MID-qE	ACGACTCCACTCACGGTAGATA	qPCR for transcripts
R_TDH3_MID-qE	CATGGCAAGTTAGCTGGGTCTCT	qPCR for transcripts
F_TDH3_3UTR-qE	AGTTGTCGACTTGGTTGAAC	qPCR for transcripts
R_TDH3_3UTR-qE	GAAACTAAGTCATAAAGCTATAAAAA	qPCR for transcripts
F_POR1_5UTR-qE	AACACGAAACAGCCAAGC	qPCR for transcripts & ratio
R_POR1_5UTR-qE	TGGAGATATCGCTGTAAACTGG	qPCR for transcripts & ratio
F_POR1_TLS-qE	TCTCCTCCAGTTTACAGCGA	qPCR for transcripts
R_POR1_TLS-qE	GATAGAAATCCTTGTTCAATAG	qPCR for transcripts
F_POR1_MID-qE	CTACCCCAGCTGCCTTTGAT	qPCR for transcripts
R_POR1_MID-qE	GCCGGTTTGCTTGTCATTCA	qPCR for transcripts
F_POR1_3UTR-qE	ACTCTGGGTGTCGGTTCCTC	qPCR for transcripts
R_POR1_3UTR-qE	TATATTAGATATATACGTTCAAGCGTC	qPCR for transcripts
F_SED1_5UTR-qE	TCGTAACTACAAAGACAAGCAAAAT	qPCR for transcripts & ratio

R_SED1_5UTR-qE	CTAAACCGGCAGATAATAGGACA	qPCR for transcripts & ratio
F_SED1_TLS-qE	TGAAATTATCAACTGTCCTATTATC	qPCR for transcripts
R_SED1_TLS-qE	CGGTGGAAGAAGCAGATGT	qPCR for transcripts
F_SED1_MID-qE	ACTTCCTCCTCTTCCATCTCCA	qPCR for transcripts
R_SED1_MID-qE	GCTGTGCTGGTACCGTTGTC	qPCR for transcripts
F_SED1_3UTR-qE	TTTTGTTATTCGTTTTTCACTTCT	qPCR for transcripts
R_SED1_3UTR-qE	AGACTAGACATGAAACGATACTGC	qPCR for transcripts
F_RDN5-1_qE	TTGCGGCCATATCTACCAG	qPCR internal standards
R_RDN5-1_qE	GATTGCAGCACCTGAGTTTC	qPCR internal standards
Fw_NPY1	GTTAGTTGAGCATATGTCCACTGCTGTGAC	npy1 cloning
Rev_NPY1	GTGAAAAGGATCCCTATGCTGAAGCACGCC	npy1 cloning
Fw_NPY1m	GCTTGTATCAGAGAAATATGGGAGCAGACAGGCA TTTCATGC	npy1 mutant cloning
Rv_NPY1m	CTCCTCTATGGTTTCTGATGGCTCCATAAAACCTG CTATTG	npy1 mutant cloning
F_T7_NAD-TDH3	AATAATACGACTCACTATAAAGAACTTAGTTTCGAA TAAACACAC	NPY1 kinetics
R_NAD-TDH3	GCAATTCTCATGACCAATCTACCGAT	NPY1 kinetics
F_CCT4_5UTR-qE	GTGTGCAAGACGGGTAAAGGAG	qPCR for ratio
R_CCT4_5UTR-qE	CAACAGAACGTGCAGCGATG	qPCR for ratio
F_TGL5_5UTR-qE	ACAGCACAAGGAAGACGGT	qPCR for ratio
R_TGL5_5UTR-qE	GAAGACAAATCGGATATGAGCAG	qPCR for ratio
F_RPS5_5UTR-qE	AAGAGACTAGAAATAACCGACCAT	qPCR for ratio
R_RPS5_5UTR-qE	CTTCTTCTGGAATTGGAGTAGCC	qPCR for ratio
F_CCW12_5UTR-qE	GAAATTAATCTTCTGTCATTCGCTT	qPCR for ratio
R_CCW12_5UTR-qE	ACAGACGTGGTCTTCACAAG	qPCR for ratio
F_PDA1_5UTR-qE	ATTTGTGCCAATGCTTGCTGCT	qPCR for ratio
R_PDA1_5UTR-qE	CATGGTACGAACATGACCTATCCTG	qPCR for ratio
F_TIM10_5UTR-qE	AGCAAAAGAAGGAACTATCATACTTAG	qPCR for ratio
R_TIM10_5UTR-qE	CAGCTCACCCTCGGAATAAGAAG	qPCR for ratio
F_TMH11_5UTR-qE	CGAAGTTTGAGAGAAAGGAACA	qPCR for ratio
R_TMH11_5UTR-qE	CTAGAGATGGAATGGAGCCCT	qPCR for ratio
F_PGC1_5UTR-qE	GAGATATAAAATTGGTGTTGTTGATG	qPCR for ratio
R_PGC1_5UTR-qE	GTTTACCACCACCATACCATCG	qPCR for ratio
F_FMP52_5UTR-qE	GATTATATAGAAGAGCACAAGCAACG	qPCR for ratio
R_FMP52_5UTR-qE	CTTTCTACTATGGCCACAACTTTATC	qPCR for ratio
F_FET3_5UTR-qE	GGATAGGCATAGGAAACGAAGAG	qPCR for ratio
R_FET3_5UTR-qE	GTGATCACGGGACGGCTCTT	qPCR for ratio
F_SED1_5UTR-qE	GAGGTTGCTGCTTTGGTTATTG	qPCR for ratio
R_SED1_5UTR-qE	CTACCGACGATAGATGGGAAGAC	qPCR for ratio
F_GLK1_5UTR-L-qR	AACTCAGCTTCCGTAAACC	qPCR for switch & ratio
R_GLK1_5UTR-L-qR	ATGACCGCTCTCTCAGTGGC	qPCR for switch & ratio
F_GLK1_5UTR-S-qR	ACCACCACTAATACAACTCTATC	qPCR for switch
R_GLK1_5UTR-S-qR	TGGATGACCGCTCTCTCAG	qPCR for switch
F_MIG1_5UTR-L-qR	AAGCATTTCGAAGATAAGAG	qPCR for switch & ratio
R_MIG1_5UTR-L-qR	GTAGTATGTCGTCTCCACT	qPCR for switch & ratio
F_MIG1_5UTR-S-qR	AGTTGAGTATAGTGGAGACGACA	qPCR for switch

R_MIG1_5UTR-S-qR	GCTACTTTGGACTTGCTTTTAC	qPCR for switch
F_LSM7_5UTR-L-qR	TGTACGTGTATACTACTACAGTAAAAAAG	qPCR for switch & ratio
R_LSM7_5UTR-L-qR	TACGGAGTGTTGCTGATGC	qPCR for switch & ratio
F_LSM7_5UTR-S-qR	GAGAGCAGCACTTTGTTTACTACAC	qPCR for switch
R_LSM7_5UTR-S-qR	AAGGAAGGTTATTGCCACGGAAG	qPCR for switch
F_RSM10_5UTR-L-qR	AACCGTGCAAAATCAGAAAAG	qPCR for switch & ratio
R_RSM10_5UTR-L-qR	GGCGCGTCGACTGTGTTC	qPCR for switch & ratio
F_RSM10_5UTR-S-qR	GCCAATGCTTAGAAATACCAT	qPCR for switch
R_RSM10_5UTR-S-qR	AACTTTAAAGGAGCATAGTAGACTG	qPCR for switch
F_TDH3_5UTR-L-qR	AGAACTTAGTTTCGAATAAACACACA	qPCR for switch
R_TDH3_5UTR-L&S-qR	CAATTCTCATGACCAATCTACC	qPCR for switch
F_TDH3_5UTR-S-qR	AACACACATAAACAAACAAAATG	qPCR for switch
F_POR1_5UTR-L-qR	AAGCGTACCCAAAGCAAAAAT	qPCR for switch
R_POR1_5UTR-L&S-qR	CTGGGGTAGCATGATAGAAATC	qPCR for switch
F_POR1_5UTR-S-qR	TCAAACCAACCTCTCAACAATG	qPCR for switch
F_SED1_5UTR-L-qR	AACTACAAAGACAAGCAAAAT	qPCR for switch
R_SED1_5UTR-L-qR	TCGGTGGAAGAAGCAGATG	qPCR for switch
F_SED1_5UTR-S-qR	CAAAATAAAATACGTTCGCTCTA	qPCR for switch
R_SED1_5UTR-S-qR	GAAGTGACATCGGTGGAAGAAG	qPCR for switch
F_GLK1_5UTR-L-gN	AGCAAAGCTTTAATACGACTCACTATAAACTCAGC TTCCGTAAACC	PCR from genome
F_GLK1_5UTR-S-gN	AGCAAAGCTTTAATACGACTCACTATAACTAATACA ACTCTATCATAC	PCR from genome
R_GLK1_5UTR-L&S-gN	TGTGTAAGTCGTCGAATG	PCR from genome
F_MIG1_5UTR-L-gN	AGCAAAGCTTTAATACGACTCACTATA AAGAAAGCCCGGTAAAG	PCR from genome
F_MIG1_5UTR-S-gN	AGCAAAGCTTTAATACGACTCACTATAAGAGTTGA GTATAGTGGAGACG	PCR from genome
R_MIG1_5UTR-L&S-gN	TCATTGGATATGGGCTT	PCR from genome
F_LSM7_5UTR-L-gN	AGCAAAGCTTTAATACGACTCACTATAATGTACGT GTATACTACTACAGTA	PCR from genome
F_LSM7_5UTR-S-gN	AGCAAAGCTTTAATACGACTCACTATAAGAGCAGC ACTTTGTTTAC	PCR from genome
R_LSM7_5UTR-L&S-gN	ATACGGAGTGTTGCTGAT	PCR from genome
F_RSM10_5UTR-L-gN	AGCAAAGCTTTAATACGACTCACTATAAACCGTGC AAAATC	PCR from genome
R_RSM10_5UTR-L-gN	AACATATGCAATGGTATTTCTAAGC	PCR from genome
F_RSM10_5UTR-S-gN	AGCAAAGCTTTAATACGACTCACTATAATTGGCCA ATGCTTAGAAATACCATTGCAT	PCR from genome
R_RSM10_5UTR-S-gN	ATGCAATGGTATTTCTAAGCATTGGCCAATTATAGT GAGTCGTATTAAAGCTTTGCT	PCR from genome
F_TDH3_5UTR-gN	AGCAAAGCTTTAATACGACTCACTATAAAGAACTTA GTTTCGAATAAACAC	PCR from genome
R_TDH3_5UTR-gN	CGTTAATAGCAACTCTAACCAT	PCR from genome
F_POR1_5UTR-gN	AGCAAAGCTTTAATACGACTCACTATAAGCGTACC CAAAGC	PCR from genome
R_POR1_5UTR-gN	TCGCTGTAAACTGGAGG	PCR from genome
F_SED1_5UTR-L-gN	AGCAAAGCTTTAATACGACTCACTATAAACTACAAA GACAAGCAAAAT	PCR from genome
F_SED1_5UTR-S-gN	AGCAAAGCTTTAATACGACTCACTATAAAGCAAAAT AAAATACGTTCG	PCR from genome
R_SED1_5UTR-L&S-gN	ATAGGACAGTTGATAATTTCAT	PCR from genome
F_Linker_Luc2	ATGTTATGGAAGATGCCAAAAACAT	PCR for Luc2

R_Luc2_polyA_HindIII	AAGCTTTTTTTTTTTTTTTTTTTTTTTTTTTTTTTTACA CGGCGATCT	PCR for Luc2 & IVT template
F_HindIII_T7_Renilla	AGCAAAGCTTTAATACGACTCACTATAATGACTTC GAAAGTTTATGATCC	PCR for Renilla
R_Renilla_polyA_HindIII	AAGCTTTTTTTTTTTTTTTTTTTTTTTTTTTTTTTTATT GTTCATTTTTGAGAACTCG	PCR for Renilla & IVT template
Bridge_Region_GLK1	ACAAGATGTCATTCGACGACTTACACAATGTTATG GAAGATGCCAAAAACATTAAG	PCR for 5UTR-mRNA
Bridge_Region_MIG1	TAGCCATGCAAAGCCCATATCCAATGAATGTTATG GAAGATGCCAAAAACATTAAG	PCR for 5UTR-mRNA
Bridge_Region_LSM7	CAAAAAAACATGCATCAGCAACACTCCGTATATGT TATGGAAGATGCCAAAAACATTAAG	PCR for 5UTR-mRNA
Bridge_Region_RSM10	GGCCAATGCTTAGAAATACCATTGCATATGTTATG GAAGATGCCAAAAACATTAAG	PCR for 5UTR-mRNA
Bridge_Region_TDH3	CAAACAAAATGGTTAGAGTTGCTATTAACGATGTTA TGGAAGATGCCAAAAACATTAAG	PCR for 5UTR-mRNA
Bridge_Region_POR1	CTCAACAATGTCTCCTCCAGTTTACAGCGAATGTT ATGGAAGATGCCAAAAACATTAAG	PCR for 5UTR-mRNA
Bridge_Region_SED1	CTATTAAGATGAAATTATCAACTGTCCTATATGTTA TGGAAGATGCCAAAAACATTAAG	PCR for 5UTR-mRNA
Luc2_inner_Sanger_F1	ATAGCAAGACCGACTACCAG	Sanger Sequencing
Luc2_inner_Sanger_F2	CTTCGGCAACCAGATCATCC	Sanger Sequencing
Luc2_inner_Sanger_R1	GTGGCAAATGGGAAGTCACG	Sanger Sequencing
Luc2_inner_Sanger_R2	TTTACCGTGTTCCAGCACGACG	Sanger Sequencing
F_5END_plasmid1	TGTGCTGGAATTCGGCTTG	PCR for IVT template
F_5END_plasmid2	CAGGAGTGTGTCTGTCTCCATG	PCR for IVT template
DNAzyme_Luc2	AGTGGGTAGAAGGCTAGCTACAACGAGGCGCTG	mRNAs NAD-ratio
F_Luc2_Mid_qE	AGACCGACTACCAGGGCTTCC	qPCR for mRNA copy
R_Luc2_Mid_qE	TCAGGGCGATGGTTTTGTCC	qPCR for mRNA copy
F_Luc2_3END_qE	GCCTGCCCGACGACGATG	qPCR for mRNA copy
R_Luc2_3END_qE	GCAGCTTCTTGGCGGTTGTAAC	qPCR for mRNA copy
F_RNAI_SI	ACAGTATTTGGTATCTGCGCT	qPCR for NAD-RNA promoter
R_RNAI_SI	AGCGGTGGTTTGTTTGC	qPCR for NAD-RNA promoter
F_RNAIII_SI	GTGATGGAAAATAGTTGATGAGT	qPCR for NAD-RNA promoter
R_RNAIII_SI	GCCATTGAAATCACTCCTTCC	qPCR for NAD-RNA promoter
F_TDH3_sfGFP	CACACATAAACAAACAAAATGGTTAG	qPCR for NAD-RNA promoter
R_TDH3_sfGFP	TATGTCCGTTTACATCTCCGTC	qPCR for NAD-RNA promoter
F_TDH3_5UTR_15ntCDS_SacI	AGAGCTCAGTTTATCATTATCAATACTGCCATTTC	PCR from genome
R_TDH3_5UTR_15ntCDS_XbaI	TTCTAGAAGCAACTCTAACCATTTTGTTTG	PCR from genome
Mutagenesis_p-1A_F	CACAAAGAACTTAGTTTCGAATAAAC	YAAG promoter mutation
Mutagenesis_p+2T_F	CACCATGAACTTAGTTTCGAATAAAC	YAAG promoter mutation
Mutagenesis_p+3T_F	CACCAATAACTTAGTTTCGAATAAAC	YAAG promoter mutation
Mutagenesis_p+459T_F	CACCA AGTTCTTTGTTTCGAATAAAC	YAAG promoter mutation
Mutagenesis_R	TTTTAAAACTAAAAAAAAGACTAACTATAAAAGTAG AATTTAAG	YAAG promoter mutation
F_sfGFP_BamHI	TGGATCC ATGTCCAAGGGTGAAGAG	PCR for sfGFP
R_sfGFP_HindIII	CAAGCTTCTTATAAAGCTCGTCCATTCC	PCR for sfGFP

Supplementary Methods:

Proteomics Sample Preparation and TMT Labeling.

Yeast cells were cultured in 100 mL YPD medium and collected at OD_{600} 0.8. The cells (biological triplicates) were washed twice with ice-cold dH_2O then twice with ice-cold PBS. The pelleted cells were then lysed by passing them twice through a French press (~0.69 kbar) in 2 mL protein lysis buffer (50 mM Tris-HCl (pH 7.5), 100 mM NaCl, 1 mM EDTA, 1 mM PMSF, 1 µg/mL leupeptin, 1 µg/mL pepstatin A). The lysate was centrifuged at 25,000 g, 4 °C, 20 min. The supernatant was collected and subsequently flash-frozen in liquid nitrogen and stored at -80 °C until all samples were ready. The protein concentration was measured by BCA assay. Reduction of disulfide bonds in cysteine-containing proteins was performed using 10 mM dithiothreitol (56 °C, 30 min, in 50 mM HEPES, pH 8.5). Reduced cysteines were alkylated with 20 mM 2-chloroacetamide (room temperature, in the dark, 30 min, 50 mM HEPES, pH 8.5). Samples were prepared following the SP3 protocol [32] and subsequently trypsin (sequencing grade) was added in an enzyme to protein ratio of 1:50 for overnight digestion at 37 °C. Peptides were labelled using the TMT10plex [33] Isobaric Label Reagent, according the manufacturer's instructions. For further sample clean up, an OASIS HLB µElution Plate (Waters) was used. Offline high pH reverse phase fractionation was carried out on an Agilent 1200 Infinity high-performance liquid chromatography system, equipped with a Gemini C18 column (3 µm, 110 Å, 100 x 1.0 mm, Phenomenex) [34].

Proteomics Mass Spectrometry Data Acquisition and Analysis. An UltiMate 3000 RSLC nano LC system (Dionex) was fitted with a trapping cartridge (µ-Precolumn C18 PepMap 100, 5 µm, 300 µm i.d. x 5 mm, 100 Å) and an analytical column (nanoEase M/Z HSS T3 column 75 µm x 250 mm C18, 1.8 µm, 100 Å, Waters). Trapping was carried out with a constant flow of solvent A (0.1% formic acid in water) at 30 µL/min onto the trapping column for 6 minutes. Subsequently, peptides were eluted via the analytical column with a constant flow of 0.3 µL/min with an increasing percentage of solvent B (0.1% formic acid in acetonitrile) from 2% to 4% in 4 min, from 4% to 8% in 2 min, followed by 8% to 28% for a further 96 min, and finally from 28% to 40% in another 10 min. The outlet of the analytical column was coupled directly to a QExactive plus (Thermo Scientific) mass spectrometer using the proxeon nanoflow source in positive ion mode.

The peptides were introduced into the QExactive plus via a Pico-Tip Emitter 360 µm OD x 20 µm ID; 10 µm tip (New Objective) and an applied spray voltage of 2.3 kV. The capillary temperature was set to 320 °C. A full mass scan was acquired with a mass range from 350 to 1400 m/z in profile mode in the FT with a resolution of 70000. The filling time was set to the maximum of 100 ms with a limitation of $3x10^6$ ions. Data-dependent acquisition (DDA) was performed with the resolution of the Orbitrap set to 35000, with a fill time of 120 ms and a limitation of 2×10^5 ions. A normalized collision energy of 32 was applied. A loop count of 10 with count 1 was used and a minimum AGC trigger of 2e2 was set. A dynamic exclusion time of 30 s was used. The peptide match algorithm was set to 'preferred' and charge exclusion 'unassigned', charge states 1, 5 - 8 were excluded. MS2 data was acquired in profile mode.

IsobarQuant [35] and Mascot (v2.2.07) were used to process the acquired data, which was searched against a Uniprot *S. cerevisiae* proteome database (UP000002311) containing common contaminants and reversed sequences. The following modifications were included into the search parameters: Carbamidomethyl (C) and TMT10 (K) (fixed modification), Acetyl (N-term), Oxidation (M) and TMT10

(N-term) (variable modifications). For the full scan (MS1) a mass error tolerance of 10 ppm and for MS/MS (MS2) spectra of 0.02 Da was set. Further parameters were set: Trypsin as protease with an allowance of maximum two missed cleavages: a minimum peptide length of seven amino acids; at least two unique peptides were required for a protein identification. The false discovery rate on peptide and protein level was set to 0.01.

The protein.txt – output file of IsobarQuant was analyzed using an R script. As a quality control filter, only proteins which were quantified with at least 2 unique peptides were used (2256 out of 6049 proteins remained). The signal_sum columns were annotated according to the experimental conditions. Batch-effects were removed with the limma (v3.38.3) package and subsequently the data was normalized using vsn (v3.50.0). limma was used again to test for differentially expressed genes between wild type and *npy1*Δ. Proteins were annotated as a hit with a fold-change bigger 50% and a false discovery rate smaller 5% and as a candidate with a fold change bigger 40% and a false discovery rate smaller 20%.

Flow Cytometry Data Acquisition and Analysis. Cells, in biological triplicates, were grown in low fluorescence synthetic complete medium lacking leucine to mid log phase. Flow cytometry (FCM) of yeast cells expressing GFP from p415-based plasmids was performed on a BD FACSCanto™ II (BD Bioscience) equipped with a 488-nm laser and a combination of 502-nm long-pass and 530/30-nm band pass emission filters for GFP detection. Total 300000 events were measured for data analysis using Flowing Software. Events of single cells were isolated. Then the events of fluorescence background were removed. Fluorescence intensity of remained events was analyzed.

Plasmid Construction. Npy1: the open reading frame (ORF) of *NPY1 gene* was PCR-amplified from *S. cerevisiae* (BY4742 strain) gDNA, and NdeI and BamHI restriction sites introduced using the respective primers (Fw_NPY1 and Rev_NPY1, Table S1). The PCR products were then purified using the QIAquick PCR Purification Kit (QIAGEN) and digested with NdeI and BamHI-HF. The digested amplicons were subsequently ligated into a NdeI- and BamHI- digested pET-28a (+) plasmid (Novagen) by T4 DNA ligase, which is subsequently referred to as pET-28a-NPY1.

Mutant Npy1 (E276Q): For site-directed mutagenesis, primers encoding the desired alterations as mismatches (Fw_NPY1m and Rev_NPY1m, Table S1) were used to modify the plasmid pET-28a-NPY1, under standard PCR conditions, to obtain pET-28a-NPY1(E276Q) mutant.

The plasmids pET-28c-NudC, pET-28c-NudC-V157A-E174A-E177A-E178A (NudC-M1) were available as lab-prepared stock.

mRNA for *in vitro* translation: The cloning procedure to obtain template mRNA sequences of interest had three distinct steps. Firstly, the 5' UTR and 22 nt of the coding sequence (CDS) of the gene were PCR-amplified from *S. cerevisiae* (BY4742 strain) gDNA using the corresponding primers (Table S1). Secondly, the firefly luciferase sequence (1653 bp, same as from pGL4.10 [Luc2] Vector (Promega)) and the *Renilla reniformis* luciferase sequence (936 bp, same as from pRL-null Vector (Promega)) were cloned from the plasmid pBK77N_hybSV40_Luc2_empty_BGH_*Renilla*_CMV and pFK-DVs-R2A, respectively, using dedicated primer pairs (Fw_Luc2, Rev_luc2, Fw_*Renilla* and Rev_*Renilla* primers; Table S1). Thirdly, the T7 promoter sequence containing a HindIII site was fused with 5'-terminal mRNA sequence, firefly luciferase sequence, and a poly$(A)_{30}$ tail sequence by corresponding, employing appropriate primers (Fw_5UTR, bridge_region and Rev_Luc2_polyA; Table S1) with two rounds of PCR amplification. PCR products were purified by 0.8% agarose gel electrophoresis.

Afterwards, 0.02 µM PCR amplicon was phosphorylated by 15 U T4 PNK with 1X T4 DNA Ligase Buffer (Thermo Scientific) in an overall volume of 20 µL. 540 fmol linear vector pDisplay-AP-CFP-TM was digested by EcoRV-HF, then dephosphorylated by 1 U Fast AP Thermosensitive Alkaline Phosphatase (Thermo Scientific) in 1X Fast AP Buffer in a total reaction volume of 20 µL. The 15 fmol phosphorylated PCR product was subsequently ligated with 5 fmol dephosphorylated linear vector by 30 U T4 DNA ligase in a 20 µL reaction at 20°C for 1 h.

YAAG promoter: The plasmid backbone used here was a derivative of the p415GPD vector [36]. The *TDH3* gene promoter and 15 nt of the CDS were obtained from *S. cerevisiae* gDNA by standard PCR amplification, using corresponding primers (F_TDH3_5UTR_15ntCDS_SacI and R_TDH3_5UTR_15ntCDS_XbaI; Table S1). The p415GPD plasmid and the generated PCR products were digested with SacI-HF and XbaI and subsequently ligated. Next, sequence superfolder GFP (sfGFP) was amplified from pMaM5 plasmid under standard PCR conditions, using appropriate primers (F_sfGFP_BamHI and R_sfGFP_HindIII; Table S1). These sfGFP-encoding amplicons and the partially assembled genetic constructs, described above, were again digested with BamHI-HF and HindIII-HF and ligated together as the pOri plasmid. Plasmids carrying mutations at the positions p-1, p+2, p+3, p+459 were generated by PCR using dedicated primer pairs, separately (Mutagenesis_R, Mutagensis_p-1A_F, Mutagensis_p+2T_F, Mutagensis_p+3T_F, and Mutagensis_p+459T_F, Table S1). Correct insert sequences of all plasmids were further confirmed by Sanger sequencing.

gDNA Extraction. The gDNA isolation procedure was based on a published method [37]. 1.5 mL of the yeast overnight culture (BY4742 strain) were pelleted and resuspended in 200 µL lysis buffer, containing 2% Triton X-100, 1% SDS, 100 mM NaCl, 10 mM Tris-HCl (pH 8.0), 1 mM EDTA (pH 8.0). Resuspended cells were kept at -80 °C for 15 min and then directly heated to 95 °C for 1 min. Samples were subjected to two additional freeze-thaw cycles, as described. The mixture was then vortexed for 30 s. Subsequently, 200 µL chloroform was added and the mixture vortexed for 2 min at RT before centrifugation. Then the aqueous layer from the centrifugation was transferred into 400 µL ethanol (ice-cold). The solution was incubated at RT for 5 min. Then the solution was centrifuged at 20,000 g for 10 min at RT. The supernatant was collected and dried under vacuum. The gDNA pellet was then resuspended in 20 µL TE buffer (10 mM Tris-HCl (pH 8.0), 1 mM EDTA (pH 8.0)).

Protein Expression and Purification. *ADPRC*: The *Pichia pastoris* GS115 pPICZαA/CYCLASE-2 strain was a gift from H.C. Lee. The protein expression and purification was performed as described previously with minor modifications [11]. First, *P. pastoris* was cultured grown on YPD agar plates, containing 100 µg/mL Zeocin. Then, single colonies were used to inoculate 10 mL liquid YPD medium, and subsequently cultured in a final volume of 500 mL YPD medium, until the optical density of the cells reached its plateau phase at about 50 mg cell pellet per mL medium. Next, the YPD medium was replaced by 500 mL BMMY medium (1% yeast extract, 2% peptone, 100 mM K_2HPO_4/KH_2PO_4 (pH 6.0), 1.34% (w/v) yeast nitrogen base with ammonium sulfate without amino acids, 0.4% mg/L biotin, 0.5% (v/v) methanol). Following the exchange of growth medium and after additional 24 h, as well as 48 h, of culturing the yeast in BMMY medium, 2.25 mL methanol were added. The supernatant of thus treated cultures was then collected, after overall 72 h, by centrifugation at 4 °C, 1000g, 10 min. The 500 mL of supernatant were filtered through a 0.45 µm filter and then concentrated employing Amicon Ultra-15 mL Centrifugal Filter Units 10 kDa, ultimately yielding 25 mL of concentrate. Contained proteins were dialyzed overnight against 1 L dialysis buffer, containing 50 mM NaOAc (pH 5.0), using a 5 kDa cut-off membrane (Carl Roth). Dialysis buffer was exchanged regularly with fresh 1 L dialysis buffer. Dialyzed protein solution was then loaded on 2X HiTrap SP HP 1mL columns at a

flowrate of 1 mL/min on an FPLC system (Bio-Rad). ADRPC was eluted by applying a salt gradient from 50 mM NaOAc (pH 4.0) to 50 mM NaOAc (pH 4.0), 1M NaCl at 0.75 mL/min. The fractions that contained the ADPRC band upon SDS-PAGE analysis were pooled. The cyclase activity was determined conducting an NGD fluorometric assay. Briefly, ADPRC was subjected to serial dilution ranging from 0.2 ng/µL to 1.2 ng/µL, with constant amount of 60 µM NGD in HEPES Buffer (50 mM HEPES, 5 mM $MgCl_2$, pH 7), in a total reaction volume of 20 µL. According to linear regression of the NGD kinetics, 1 U of activity was defined as 0.125 µg ADPRC that, at a concentration of 1.35 µg/mL, converted 60 mM NGD and reached a cGDPr fluorescence plateau after 130-140s (JASCO spectrophotometer, λ_{ex} = 300 nm; λ_{em} = 410 nm, high sensitivity, bandwidth 2 nm) at 25 °C [12].

Expression and affinity purification of Npy1 and Npy1(E276Q) was achieved by the following standard procedures with minor changes. Expression in *E. coli*, carrying the corresponding expression vector, was induced at an OD_{600} of ~0.7) by adding 0.1 mM IPTG. The cells were then chilled for 20 min at 4 °C and incubated at 16 °C, 150 rpm, for an additional 16 h. Cell pellets were subsequently harvested by centrifugation and washed with ice-cold dH_2O. The pelleted cells were then resuspended in HisTrap Buffer A (50 mM Tris-HCl (pH 7.8), 0.3 M NaCl, 5 mM $MgSO_4$, 5 mM 2-mercaptoethanol, 5% glycerol, 5 mM imidazole) and cells lysed by sonification. After centrifugation (37,500 g, 4°C, 30 min) of thus obtained lysates, the supernatant was filtered through a 0.45 µm filter, before loading it on a HisTrap HP 1 mL Column, using an FPLC system (Bio-Rad). The target protein was then eluted by an imidazole gradient, ranging from HisTrap Buffer A to HisTrap Buffer B, which contained an additional 500 mM imidazole. Based on SDS-PAGE analysis, fractions containing the target protein were pooled and concentrated by Amicon Ultra-15 mL Centrifugal Filter Units 10 kDa (Merck) and the HisTrap Buffer B was exchanged with Buffer G ((50 mM Tris-HCl (pH 7.5), 200 mM NaCl, 0.1 mM DTT). Further purification of Npy1 and Npy1(E276Q) by size-exclusion chromatography (SEC) was achieved on a Sephacryl 16/60 S-200 High Resolution column. The final concentration of all proteins was determined employing the Pierce BCA Protein Assay Kit and stored in 50% glycerol at -20 °C.

Supplementary references:

1 Nagalakshmi, U. *et al.* The transcriptional landscape of the yeast genome defined by RNA sequencing. *Science* **320**, 1344-1349, (2008).

2 Walters, R. W. *et al.* Identification of NAD$^+$ capped mRNAs in Saccharomyces cerevisiae. *Proc. Natl. Acad. Sci. U. S. A.* **114**, 480-485, (2017).

3 Frindert, J. *et al.* Identification, Biosynthesis, and Decapping of NAD-Capped RNAs in B. subtilis. *Cell Rep.* **24**, 1890-1901, (2018).

4 Cáhova, H., Winz, M. L., Höfer, K., Nübel, G. & Jäschke, A. NAD captureSeq indicates NAD as a bacterial cap for a subset of regulatory RNAs. *Nature* **519**, 374-377, (2015).

5 Morales-Filloy, H. G. *et al.* The 5' NAD Cap of RNAIII Modulates Toxin Production in Staphylococcus aureus Isolates. *J. Bacteriol.* **202**, e00591-00519, (2020).

6 Meijer, H. A. *et al.* Translational repression and eIF4A2 activity are critical for microRNA-mediated gene regulation. *Science* **340**, 82-85, (2013).

7 Brachmann, C. B. *et al.* Designer deletion strains derived from Saccharomyces cerevisiae S288C: a useful set of strains and plasmids for PCR-mediated gene disruption and other applications. *Yeast* **14**, 115-132, (1998).

8 Giaever, G. *et al.* Functional profiling of the Saccharomyces cerevisiae genome. *Nature* **418**, 387-391, (2002).

9 Knop, M. & Schiebel, E. Receptors determine the cellular localization of a gamma-tubulin complex and thereby the site of microtubule formation. *EMBO J.* **17**, 3952-3967, (1998).

10 Meurer, M. *et al.* Genome-wide C-SWAT library for high-throughput yeast genome tagging. *Nat Methods* **15**, 598-600, (2018).

11 Munshi, C. & Lee, H. C. High-level expression of recombinant Aplysia ADP-ribosyl cyclase in offhia pastoris by fermentation. *Protein Expr. Purif.* **11**, 104-110, (1997).

12 Winz, M. L. *et al.* Capture and sequencing of NAD-capped RNA sequences with NAD captureSeq. *Nat. Protoc.* **12**, 122-149, (2017).

13 Nübel, G., Sorgenfrei, F. A. & Jäschke, A. Boronate affinity electrophoresis for the purification and analysis of cofactor-modified RNAs. *Methods* **117**, 14-20, (2017).

14 Fischl, W. & Bartenschlager, R. High-throughput screening using dengue virus reporter genomes. *Methods Mol. Biol.* **1030**, 205-219, (2013).

15 Janke, C. *et al.* A versatile toolbox for PCR-based tagging of yeast genes: new fluorescent proteins, more markers and promoter substitution cassettes. *Yeast* **21**, 947-962, (2004).

16 Mumberg, D., Muller, R. & Funk, M. Regulatable promoters of Saccharomyces cerevisiae: comparison of transcriptional activity and their use for heterologous expression. *Nucleic Acids Res.* **22**, 5767-5768, (1994).

17 Langmead, B., Trapnell, C., Pop, M. & Salzberg, S. L. Ultrafast and memory-efficient alignment of short DNA sequences to the human genome. *Genome Biol.* **10**, R25, (2009).

18 Li, H. & Durbin, R. Fast and accurate short read alignment with Burrows-Wheeler transform. *Bioinformatics* **25**, 1754-1760, (2009).

19 Li, H. *et al.* The Sequence Alignment/Map format and SAMtools. *Bioinformatics* **25**, 2078-2079, (2009).

20 Freese, N. H., Norris, D. C. & Loraine, A. E. Integrated genome browser: visual analytics platform for genomics. *Bioinformatics* **32**, 2089-2095, (2016).

21 Anders, S., Pyl, P. T. & Huber, W. HTSeq--a Python framework to work with high-throughput sequencing data. *Bioinformatics* **31**, 166-169, (2015).

22 Love, M. I., Huber, W. & Anders, S. Moderated estimation of fold change and dispersion for RNA-seq data with DESeq2. *Genome Biol.* **15**, 550, (2014).

23 Huang da, W., Sherman, B. T. & Lempicki, R. A. Systematic and integrative analysis of large gene lists using DAVID bioinformatics resources. *Nat. Protoc.* **4**, 44-57, (2009).

24 Aibar, S., Fontanillo, C., Droste, C. & De Las Rivas, J. Functional Gene Networks: R/Bioc package to generate and analyse gene networks derived from functional enrichment and clustering. *Bioinformatics* **31**, 1686-1688, (2015).

25 Perkins, D. N., Pappin, D. J. C., Creasy, D. M. & Cottrell, J. S. Probability-based protein identification by searching sequence databases using mass spectrometry data. *Electrophoresis* **20**, 3551-3567, (1999).

26 Bailey, T. L. *et al.* MEME SUITE: tools for motif discovery and searching. *Nucleic Acids Res.* **37**, W202-208, (2009).

27 Crooks, G. E., Hon, G., Chandonia, J. M. & Brenner, S. E. WebLogo: a sequence logo generator. *Genome Res.* **14**, 1188-1190, (2004).

28 Tripathi, S. *et al.* Meta- and Orthogonal Integration of Influenza "OMICs" Data Defines a Role for UBR4 in Virus Budding. *Cell Host Microbe* **18**, 723-735, (2015).

29 Shannon, P. *et al.* Cytoscape: a software environment for integrated models of biomolecular interaction networks. *Genome Res.* **13**, 2498-2504, (2003).

30 Ritchie, M. E. *et al.* limma powers differential expression analyses for RNA-sequencing and microarray studies. *Nucleic Acids Res.* **43**, e47, (2015).

31 Huber, W., von Heydebreck, A., Sultmann, H., Poustka, A. & Vingron, M. Variance stabilization applied to microarray data calibration and to the quantification of differential expression. *Bioinformatics* **18 Suppl 1**, S96-104, (2002).

32 Hughes, C. S. *et al.* Ultrasensitive proteome analysis using paramagnetic bead technology. *Mol. Syst. Biol.* **10**, 757, (2014).
33 Werner, T. *et al.* Ion coalescence of neutron encoded TMT 10-plex reporter ions. *Anal. Chem.* **86**, 3594-3601, (2014).
34 Reichel, M. *et al.* In Planta Determination of the mRNA-Binding Proteome of Arabidopsis Etiolated Seedlings. *Plant Cell* **28**, 2435-2452, (2016).
35 Franken, H. *et al.* Thermal proteome profiling for unbiased identification of direct and indirect drug targets using multiplexed quantitative mass spectrometry. *Nat. Protoc.* **10**, 1567-1593, (2015).
36 Mumberg, D., Muller, R. & Funk, M. Yeast vectors for the controlled expression of heterologous proteins in different genetic backgrounds. *Gene* **156**, 119-122, (1995).
37 Harju, S., Fedosyuk, H. & Peterson, K. R. Rapid isolation of yeast genomic DNA: Bust n' Grab. *BMC Biotechnol.* **4**, 8, (2004).
