# Supplementary material for: Extensive 5′-surveillance guards against non-canonical NAD-caps of nuclear mRNAs in yeast
Source: Nat Commun. 2020 Nov 2;11:5508. doi: 10.1038/s41467-020-19326-3 (PMC7606564; doi:10.1038/s41467-020-19326-3)
Supplement: Supplementary file 3 — Reporting Summary [file 41467_2020_19326_MOESM3_ESM.pdf]

nature research

Corresponding author(s): Prof. Dr. Andres Jäschke

Last updated by author(s): 2020/09/07

Reporting Summary

Nature Research wishes to improve the reproducibility of the work that we publish. This form provides structure for consistency and transparency in reporting. For further information on Nature Research policies, see our Editorial Policies and the Editorial Policy Checklist.

Statistics

For all statistical analyses, confirm that the following items are present in the figure legend, table legend, main text, or Methods section.

n/a	Confirmed	
☐	☒	The exact sample size (*n*) for each experimental group/condition, given as a discrete number and unit of measurement
☒	☐	A statement on whether measurements were taken from distinct samples or whether the same sample was measured repeatedly
☐	☒	The statistical test(s) used AND whether they are one- or two-sided<br>*Only common tests should be described solely by name; describe more complex techniques in the Methods section.*
☐	☒	A description of all covariates tested
☐	☒	A description of any assumptions or corrections, such as tests of normality and adjustment for multiple comparisons
☐	☒	A full description of the statistical parameters including central tendency (e.g. means) or other basic estimates (e.g. regression coefficient) AND variation (e.g. standard deviation) or associated estimates of uncertainty (e.g. confidence intervals)
☐	☒	For null hypothesis testing, the test statistic (e.g. *F*, *t*, *r*) with confidence intervals, effect sizes, degrees of freedom and *P* value noted<br>*Give P values as exact values whenever suitable.*
☒	☐	For Bayesian analysis, information on the choice of priors and Markov chain Monte Carlo settings
☒	☐	For hierarchical and complex designs, identification of the appropriate level for tests and full reporting of outcomes
☐	☒	Estimates of effect sizes (e.g. Cohen's *d*, Pearson's *r*), indicating how they were calculated

Our web collection on statistics for biologists contains articles on many of the points above.

Software and code

Policy information about availability of computer code

Data collection	No software was used for this purpose.
Data analysis	ImageQuant-8.1, Bowtie-1.1.2, Bwa-0.7.13, samtools-0.1.13, Integrated Genome Browser, HTSeq-0.6.0, DESeq2-1.4.5, Bioconductor-2.14, David-6.7, FGNet-3.16.0, Mascot-2.2.07, MEME-5.0.3, WegLogo-3, Metascape, Cytoscape-3.7.0, limma- 3.38.3, vsn-3.50.0, Flowing Software-2.5.1

For manuscripts utilizing custom algorithms or software that are central to the research but not yet described in published literature, software must be made available to editors and reviewers. We strongly encourage code deposition in a community repository (e.g. GitHub). See the Nature Research guidelines for submitting code & software for further information.

Data

Policy information about availability of data

All manuscripts must include a data availability statement. This statement should provide the following information, where applicable:
- Accession codes, unique identifiers, or web links for publicly available datasets
- A list of figures that have associated raw data
- A description of any restrictions on data availability

NGS raw data and analyzed files are available in the GEO repository under the GEO Accession: GSE146368. Proteomics raw data and analyzed files are deposited in the ProteomeXchange Consortium via the PRIDE repository: PXD017893.

Field-specific reporting

Please select the one below that is the best fit for your research. If you are not sure, read the appropriate sections before making your selection.

☒ Life sciences ☐ Behavioural & social sciences ☐ Ecological, evolutionary & environmental sciences

For a reference copy of the document with all sections, see nature.com/documents/nr-reporting-summary-flat.pdf

Life sciences study design

All studies must disclose on these points even when the disclosure is negative.

Sample size	No specific calculation for the sample size.
Data exclusions	No data were excluded without mention.
Replication	All attempts for NGS library and proteomics construction were successful.
Randomization	No specific sample randomization for this study. The seed for programming random was 922 for analysis.
Blinding	No specific use for blinding on this study.

Reporting for specific materials, systems and methods

We require information from authors about some types of materials, experimental systems and methods used in many studies. Here, indicate whether each material, system or method listed is relevant to your study. If you are not sure if a list item applies to your research, read the appropriate section before selecting a response.

Materials & experimental systems

n/a	Involved in the study	
☒	☐	Antibodies
☐	☒	Eukaryotic cell lines
☒	☐	Palaeontology and archaeology
☒	☐	Animals and other organisms
☒	☐	Human research participants
☒	☐	Clinical data
☒	☐	Dual use research of concern

Methods

n/a	Involved in the study	
☒	☐	ChIP-seq
☐	☒	Flow cytometry
☒	☐	MRI-based neuroimaging

Eukaryotic cell lines

Policy information about cell lines

Cell line source(s)	Yeast BY4742 Strains; Yeast ESM356-1 Strains; Yeast C-SWAT mNeonGreen (mNG-I) Strains; Yeast BY4741 Strains
Authentication	Gene deletions were performed using standard PCR-based recombination methods as described (Janke et al., 2004; Sikorski and Hieter, 1989), followed by PCR-based confirmation
Mycoplasma contamination	*Confirm that all cell lines tested negative for mycoplasma contamination OR describe the results of the testing for mycoplasma contamination OR declare that the cell lines were not tested for mycoplasma contamination.*
Commonly misidentified lines (See ICLAC register)	*Name any commonly misidentified cell lines used in the study and provide a rationale for their use.*

Flow Cytometry

Plots

Confirm that:

☐ The axis labels state the marker and fluorochrome used (e.g. CD4-FITC).

☐ The axis scales are clearly visible. Include numbers along axes only for bottom left plot of group (a 'group' is an analysis of identical markers).

☒ All plots are contour plots with outliers or pseudocolor plots.

☒ A numerical value for number of cells or percentage (with statistics) is provided.

Methodology

Sample preparation	Cells, in biological triplicates, were grown in low fluorescence synthetic complete medium lacking leucine to mid log phase.
Instrument	BD FACSCantoTM II (BD Bioscience) equipped with a 488-nm laser and a combination of 502-nm long-pass and 530/30-nm band pass emission filters for GFP detection
Software	Flowing Software
Cell population abundance	Total 300000 events were measured for data analysis
Gating strategy	Events of single cells were isolated. Then the events of fluorescence background were removed. Fluorescence intensity of remained events was analyzed.

☐ Tick this box to confirm that a figure exemplifying the gating strategy is provided in the Supplementary Information.
