# Supplementary figures and images for: Extensive 5′-surveillance guards against non-canonical NAD-caps of nuclear mRNAs in yeast
Source: Nat Commun. 2020 Nov 2;11:5508. doi: 10.1038/s41467-020-19326-3 (PMC7606564; doi:10.1038/s41467-020-19326-3)

Fig. 3a

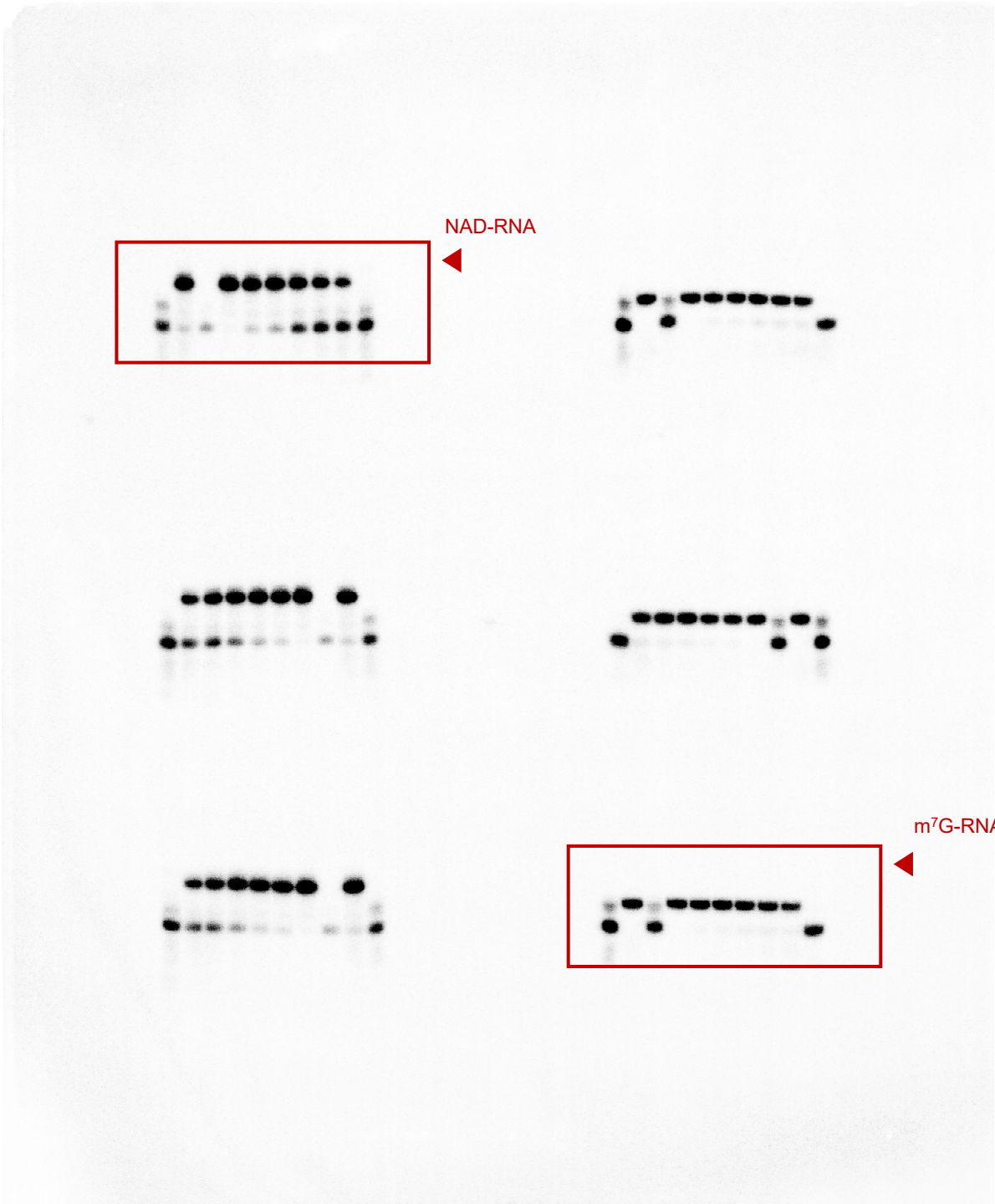

Fig. 4d

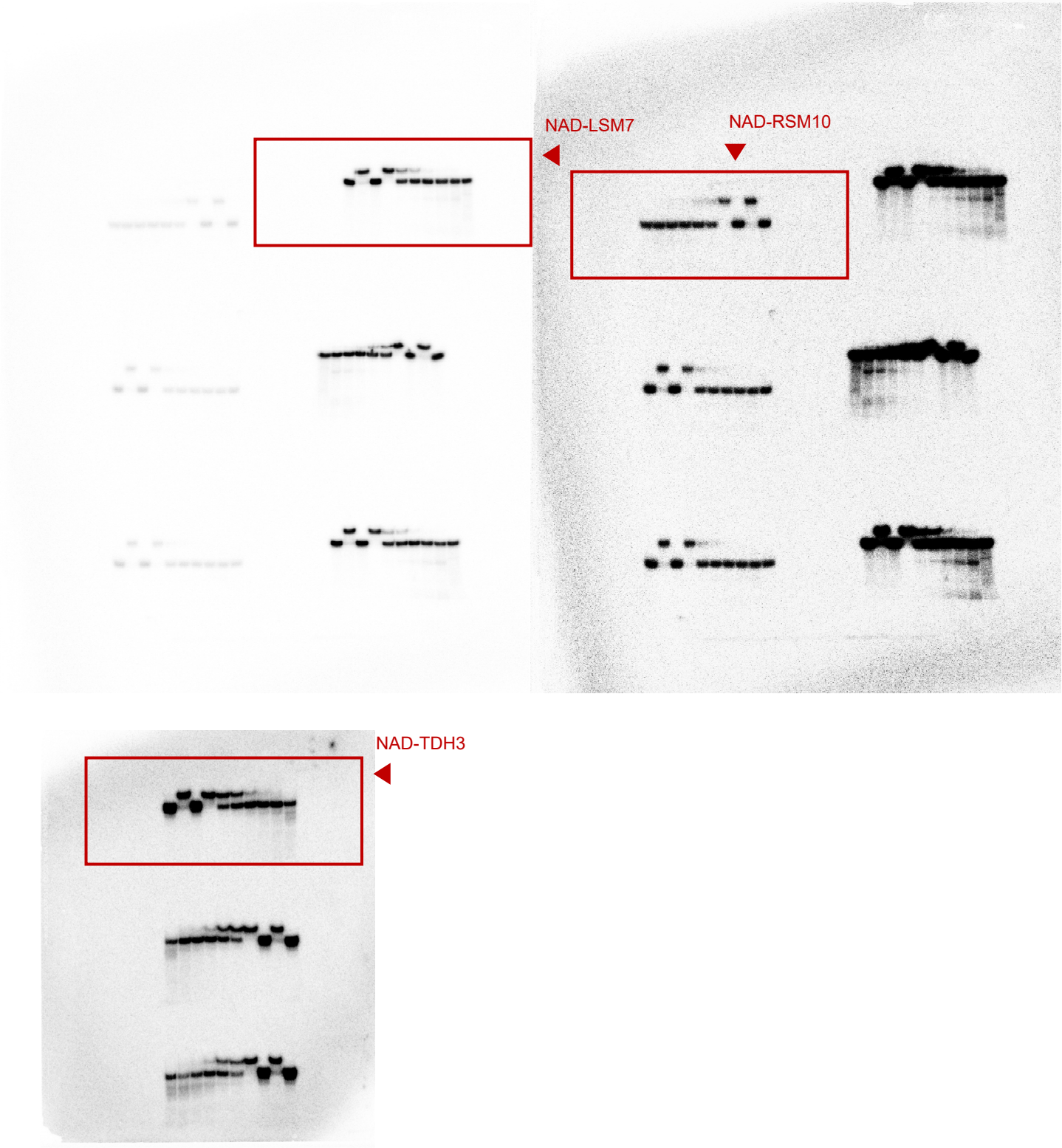

Fig. 5a

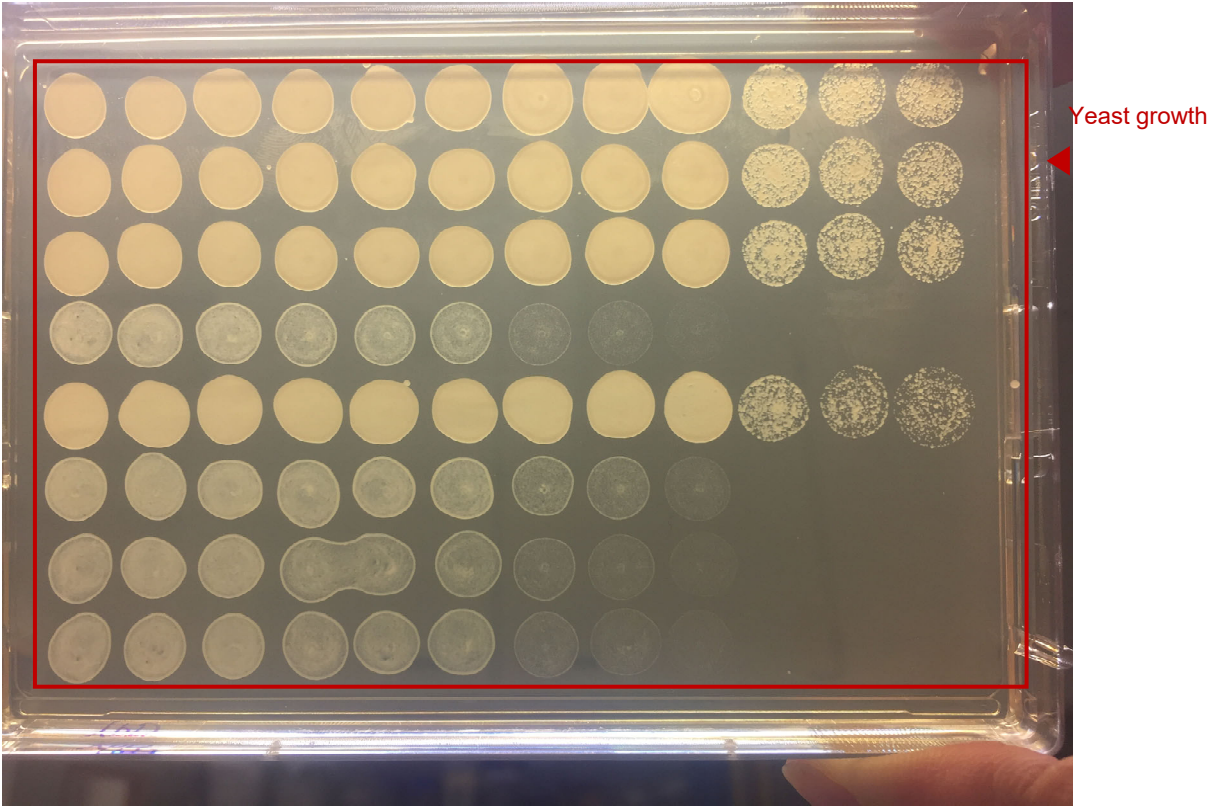

Fig. 7d

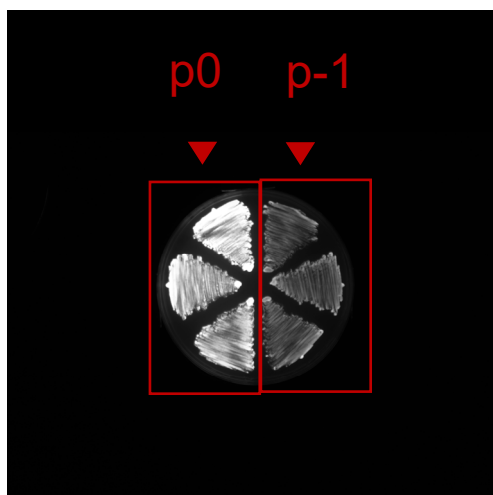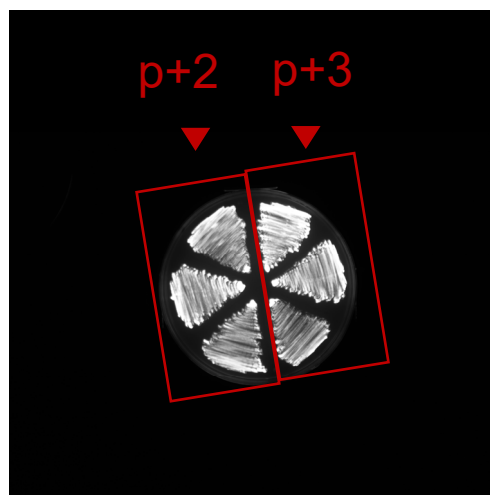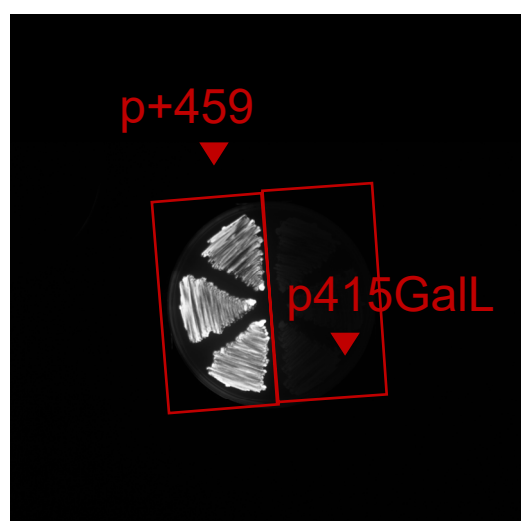

Fig. 9a

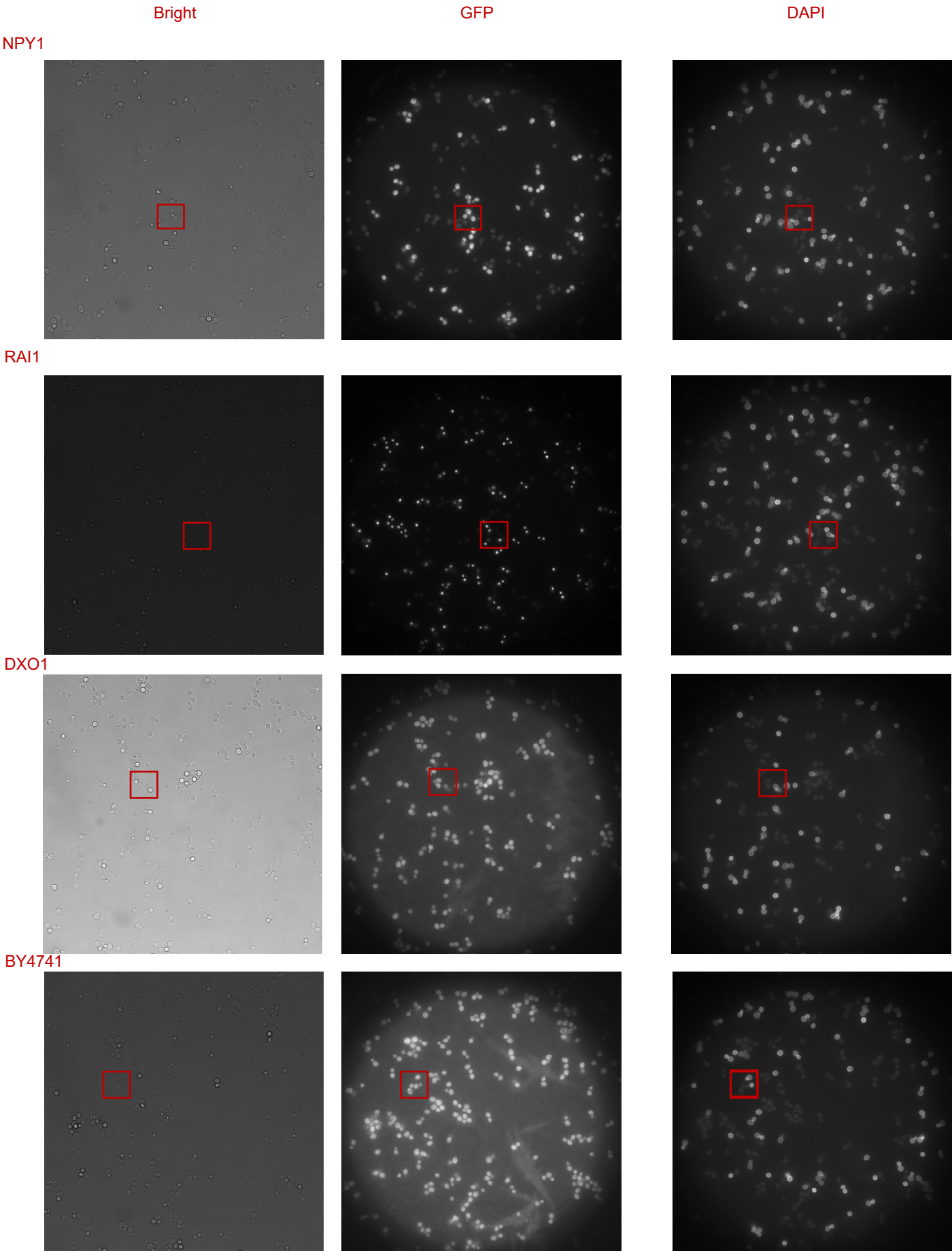

Fig. S2c

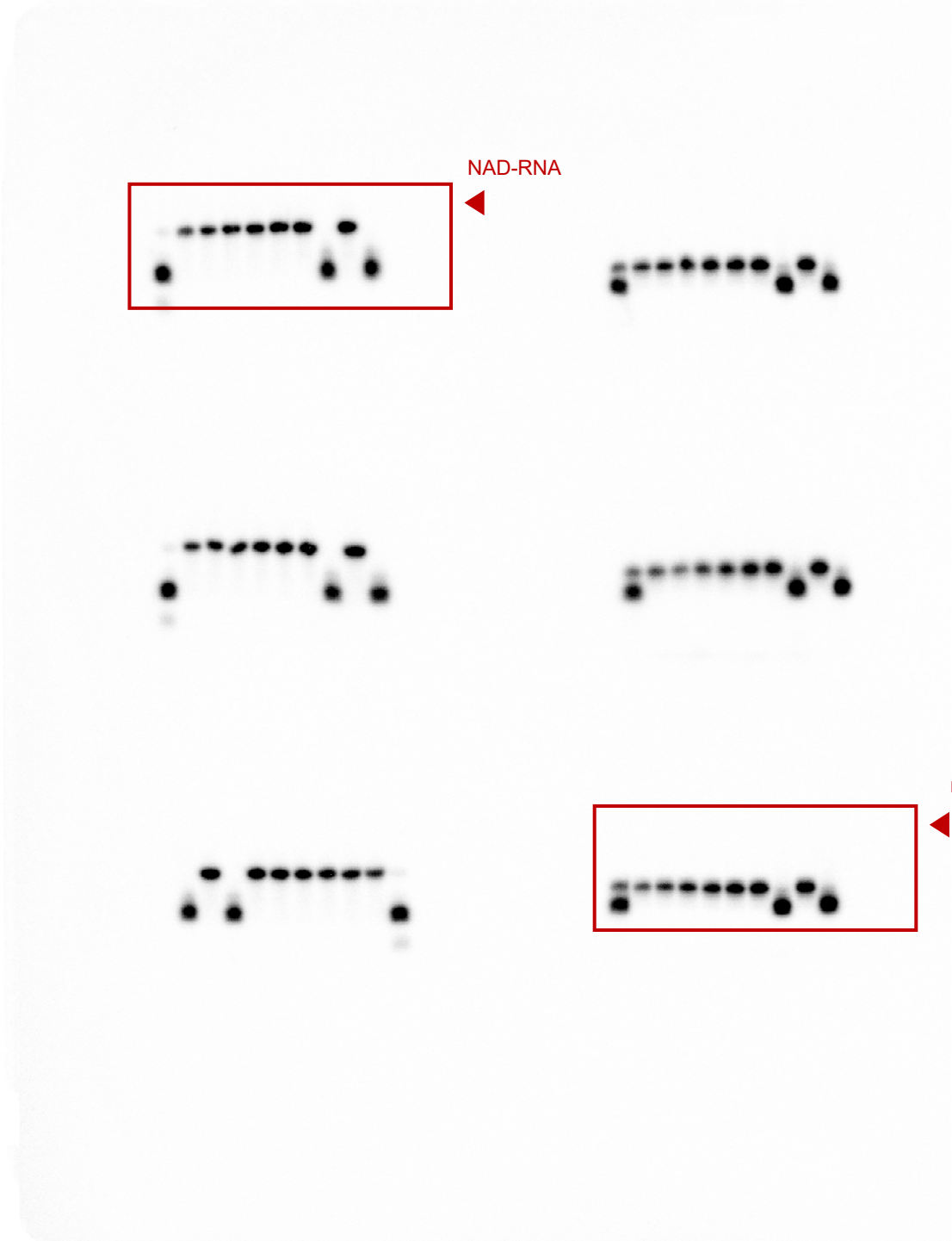

Fig. S2d

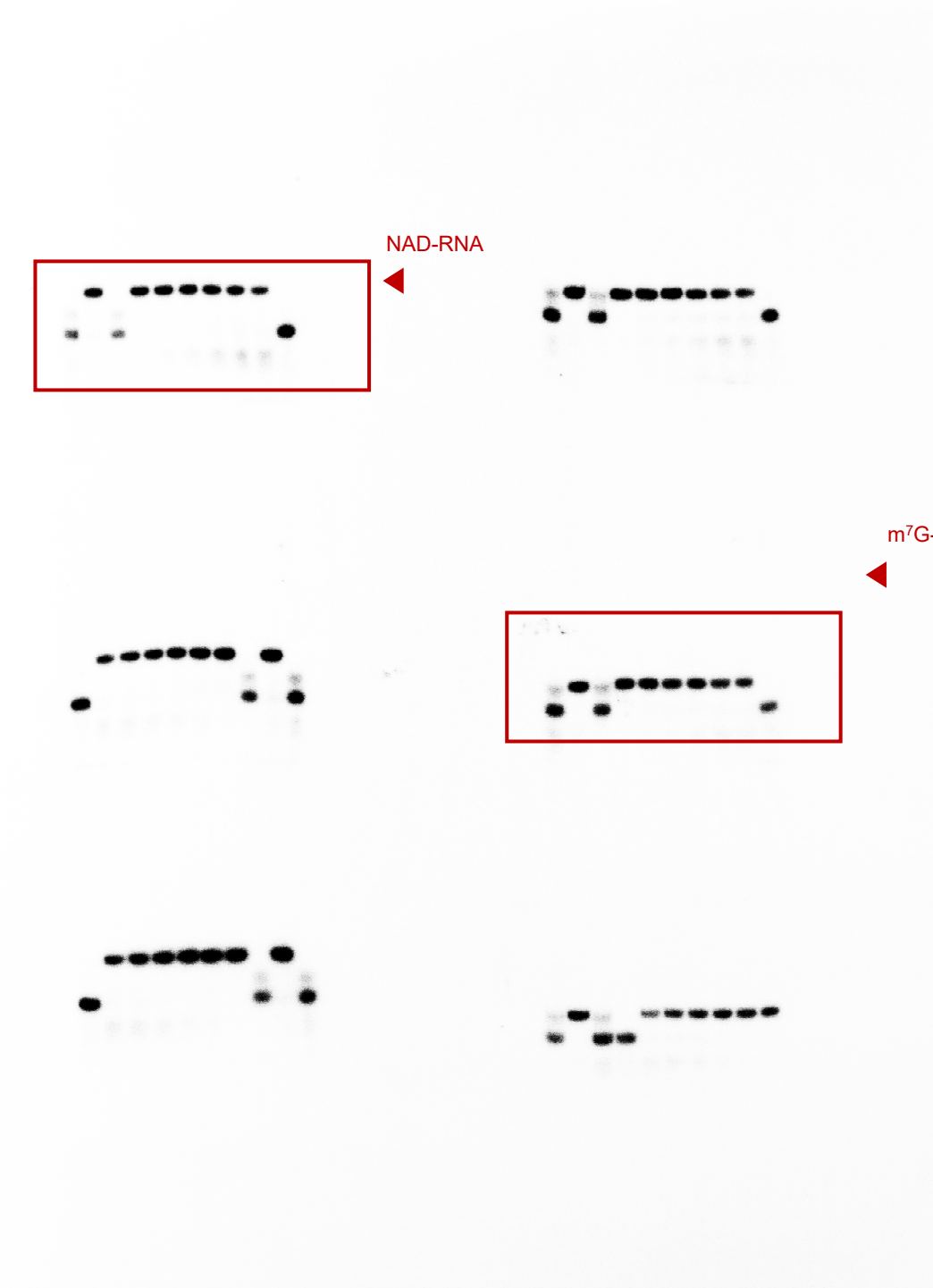

Fig. S2e

Fig. S2f

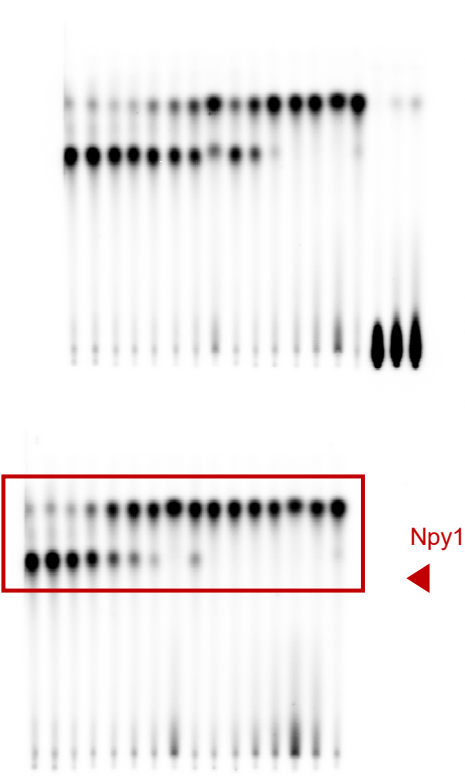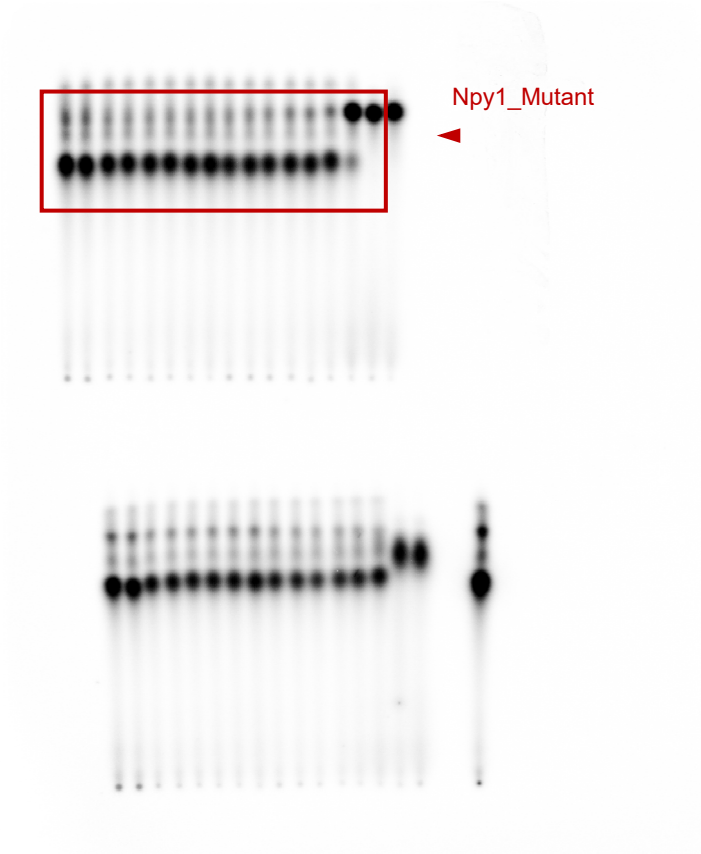

Fig. 2g

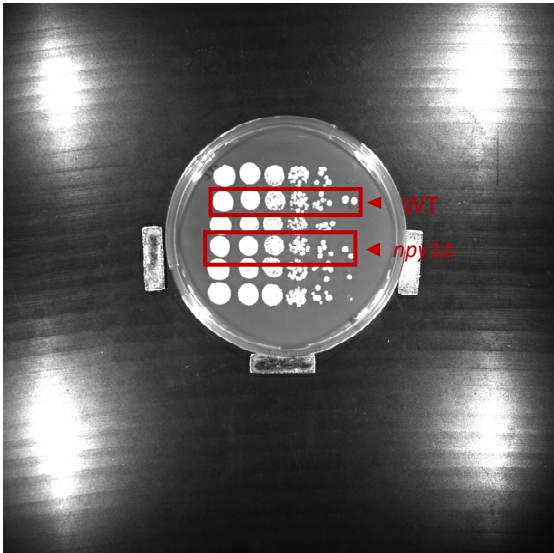

*NaCl*

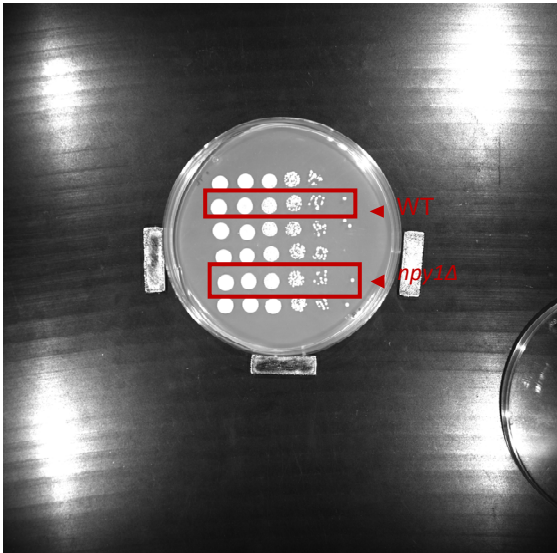

23 °C

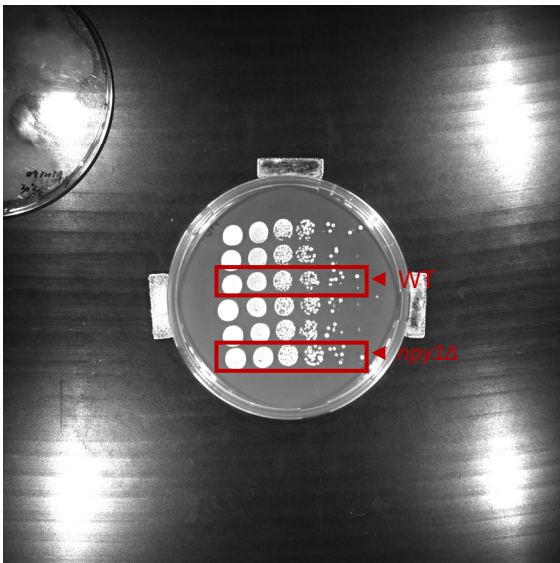

30 °C

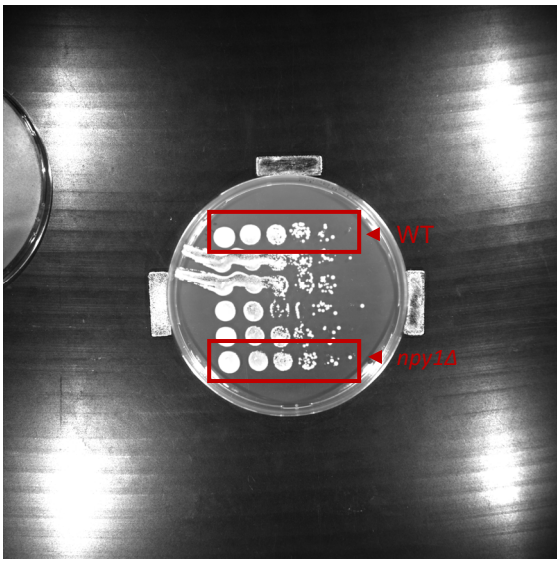

37 °C

Supplement: Supplementary file 4 — Source Data [file 41467_2020_19326_MOESM4_ESM.zip › sourceDataForImages.pdf]
